# PEDOT:PSS for Flexible and Stretchable Electronics: Modifications, Strategies, and Applications

**DOI:** 10.1002/advs.201900813

**Published:** 2019-07-30

**Authors:** Xi Fan, Wanyi Nie, Hsinhan Tsai, Naixiang Wang, Huihui Huang, Yajun Cheng, Rongjiang Wen, Liujia Ma, Feng Yan, Yonggao Xia

**Affiliations:** ^1^ Ningbo Institute of Materials Technology and Engineering Chinese Academy of Sciences Ningbo 315201 China; ^2^ Division of Materials Physics and Application Los Alamos National Laboratory Los Alamos NM 87545 USA; ^3^ Department of Applied Physics The Hong Kong Polytechnic University Hung Hom Kowloon Hong Kong 999077 China; ^4^ Center of Materials Science and Optoelectronics Engineering University of Chinese Academy of Sciences Beijing 100049 China; ^5^ School of Physics and Electronics Hunan University Changsha 410082 China

**Keywords:** conductive polymers, flexible organic/perovskite solar cells, stretchable devices, transistors

## Abstract

Substantial effort has been devoted to both scientific and technological developments of wearable, flexible, semitransparent, and sensing electronics (e.g., organic/perovskite photovoltaics, organic thin‐film transistors, and medical sensors) in the past decade. The key to realizing those functionalities is essentially the fabrication of conductive electrodes with desirable mechanical properties. Conductive polymers (CPs) of poly(3,4‐ethylenedioxythiophene):poly(styrenesulfonate) (PEDOT:PSS) have emerged to be the most promising flexible electrode materials over rigid metallic oxides and play a critical role in these unprecedented devices as transparent electrodes, hole transport layers, interconnectors, electroactive layers, or motion‐sensing conductors. Here, the current status of research on PEDOT:PSS is summarized including various approaches to boosting the electrical conductivity and mechanical compliance and stability, directly linked to the underlying mechanism of the performance enhancements. Along with the basic principles, the most cutting edge‐progresses in devices with PEDOT:PSS are highlighted. Meanwhile, the advantages and plausible problems of the CPs and as‐fabricated devices are pointed out. Finally, new perspectives are given for CP modifications and device fabrications. This work stresses the importance of developing CP films and reveals their critical role in the evolution of these next‐generation devices featuring wearable, deformable, printable, ultrathin, and see‐through characteristics.

## 1 Introduction

Wearable, flexible, and stretchable devices become the forefront of optoelectronic, sensing electronic researches.^1^, ^2^, ^3^, ^4^, ^5^, ^6^, ^7^, ^8^, ^9^, ^10^ Flexible devices offer robust performance under bending, twisting, and folding conditions, whereas stretchable devices not only require an extremely high degree of flexibility but also afford a tensile strain (ε) of at least 10%. The ε is defined as Δ*L*/*L*_0_, where *L*_0_ is the initial length of films under normal conditions, and Δ*L* is the increased length of films increased under strains. These fields give rise to considerable demands of organic solar cells (OSCs),^11^, ^12^, ^13^, ^14^, ^15^, ^16^, ^17^, ^18^, ^19^, ^20^ perovskite solar cells (PSCs),^21^, ^22^, ^23^, ^24^, ^25^, ^26^ organic light‐emitting diodes (OLEDs),^27^, ^28^, ^29^, ^30^, ^31^, ^32^, ^33^ organic thin‐film transistors (OTFTs),^34^, ^35^, ^36^, ^37^, ^38^, ^39^, ^40^, ^41^, ^42^, ^43^, ^44^ thermoelectrics,^45^, ^46^, ^47^, ^48^, ^49^ health monitors,^50^, ^51^, ^52^, ^53^, ^54^, ^55^ flexible touch sensors,^56^ and artificial intelligent robotics.^57^, ^58^, ^59^, ^60^ Wearable, flexible, and stretchable electronics provide unprecedented fashions and infinite varieties for their powerful penetrations into the world market.

**Figure 1:**
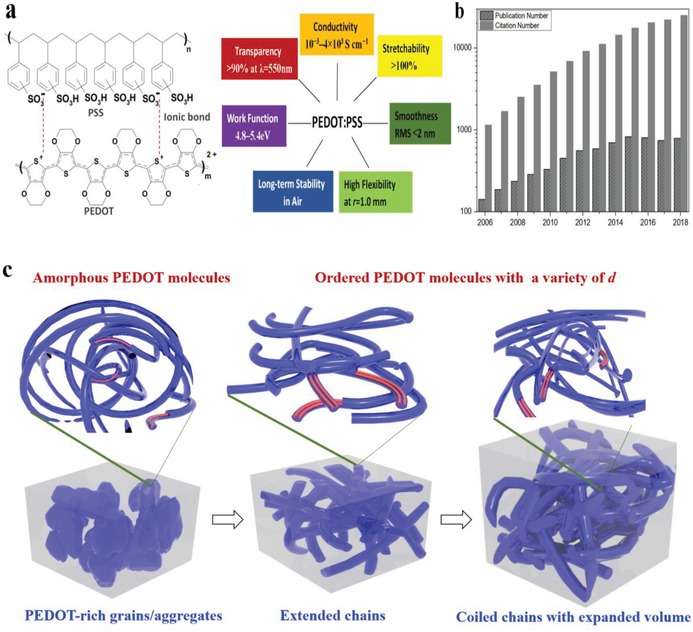
a) Chemical structures and features of PEDOT:PSS. b) Number of publications and citations sorted by year with the key word “PEDOT:PSS” indexed in the Web of Science in February 2019. c) Schematic diagram of a structural rearrangement of a typical initial film, a highly conductive film, and a highly conductive and intrinsic stretchable film of PEDOT:PSS.

However, simultaneously achieving high‐performance fluxes and mechanical compliance has been a major challenge. To enable all those functionalities, electrode materials with high mechanical flexibility and optical transparency while maintaining their electrical conductivity are the core, meanwhile the electrode fabrication techniques remains to be the biggest challenge in the research community. As one of the most classic conductive polymers (CPs), poly(3,4‐ethylenedioxythiophene):poly(styrenesulfonate) (called PEDOT:PSS, see **Figure** **1**a), because of its optical transparency at visible range, tunable electrical conductivity and work function (WF), high flexibility, stretchability, etc., is the most widely used materials for fabricating the existing photovoltaics (PVs), displays and transistors and various sensing electronics including strain‐, pressure‐, temperature‐, humid‐, and biosensors.^5^, ^37^, ^38^, ^55^, ^60^, ^61^, ^62^, ^63^, ^64^, ^65^, ^66^, ^67^, ^68^, ^69^, ^70^ As shown in Figure 1b, academic publications and citations with regard to the research on PEDOT:PSS and its applications undergo exponential growths. PEDOT:PSS is a polymer electrolyte incorporating conducting conjugated PEDOT with positive charges and insulating PSS with negative charges. Oxidized PEDOT is highly conductive but is insoluble in water; whereas insulating PSS facilitates the dispersion of PEDOT in water and enables a stabilized PEDOT configuration by Columbic attractions. The aqueous dispersions of PEDOT:PSS with deep‐blue opaque colors have been commercialized under the trade names of Baytron by Bayer AG, Clevios by Heraeus, and Orgacon by Agfa.^71^, ^72^ Among them, the PEDOT:PSS aqueous solutions (Clevios) are widely employed to prepare highly conductive and stretchable electrodes (Clevios PH500,^73^, ^74^ Clevios PH510,^75^, ^76^, ^77^ and Clevios PH1000^44^, ^78^, ^79^, ^80^, ^81^ used) and hole transport/injection layers (Clevios P VP AI4083 used^82^, ^83^, ^84^, ^85^, ^86^, ^87^) in the lab‐size electronics. Much effort has been also devoted to development of the CP inks for a variety of scalable deposition methods such as blade coating, slot die coating, shear coating, spray deposition, inkjet printing, and screen printing,^88^, ^89^, ^90^, ^91^, ^92^, ^93^, ^94^, ^95^, ^96^ which bring the CP films closer to commercial applications in cost‐effective flexible electronics. Notably, compared to other typical dispersions of Ag nanowires (NWs)^97^, ^98^, ^99^, ^100^ and carbon nanotubes (CNTs)^101^, ^102^, ^103^, ^104^, ^105^, ^106^ for printable film depositions, the PEDOT:PSS dispersions offer extremely low fabrication costs (**Table** **1**), and the CP films show the striking merits such as high uniformity and smoothness (roughness <2.0 nm) regardless of glass and plastic underlying substrates used, which have enabled monolayer interface and emitting device and transistor applications that no other materials could achieve.

**Table 1:** Costs of the solutions of the candidate materials for fabrication of flexible transparent films

Product	Merits (introduced by Sigma‐Aldrich)	Prices^a)^ [USD mL^−1^]	Concentration
PEDOT:PSS	1 S cm^−1^	163/250 = 0.65	1.3 wt%
	125 Ω sq^−1^, *T*: 90%, transparent electrodes in OPV	135/100 = 1.35	0.5–1.0 wt%
	Hole injection layers in OLED	214/100 = 2.14	2.8 wt%
	Highly conductive hole transport layer	122/25 = 4.88	0.8 wt% in H_2_O
	1500 Ω sq^−1^ (6 µm), conductive inks suitable for transistor and strain sensor	145.8/25 = 5.83	3.0–4.0 wt%
Ag NW	120–150 nm × 20–50 µm (size)	222/25 = 8.88	0.5 wt% in IPA
	115 nm × 20–50 µm	350/25 = 14.00	
	60 nm × 10 µm	418/25 = 16.72	
SWCNT	<10^3^ Ω sq^−1^, *T*: 85%	190/25 = 7.60	1.0 g mL^−1^ in H_2_O
	<600 Ω sq^−1^, *T*: 85%	216/25 = 8.64	

^a)^ Price collected from the website of Sigma‐Aldrich (January 2019).

As‐cast PEDOT:PSS films have an inherent direct current electrical conductivity (σ) of no more than 1.0 S cm^−1^;^81^, ^107^, ^108^ whereas the modified films are able to show a substantial improvement in conductivity to 2–3 orders of magnitude (the best values reach 4000 S cm^−1^) through the doping of polar solvents,^93^ strong acids,^78^, ^79^, ^80^, ^81^ ionic liquids,^109^ etc. Additionally, the CP films have a typical work function (WF: 4.8–5.4 eV) that is favored for charge transfer and injection with fast kinetics, and the films are preferably used in optoelectronic devices as *p*‐type contact layer. Furthermore, the doped PEDOT:PSS films became flexible and intrinsically stretchable,^78^, ^79^, ^80^, ^81^, ^93^, ^109^, ^110^ and thus the films were able to afford mechanical tests including impacting, shearing, bending, twisting, folding, and large tensile strains of over 100%. Here, we propose the model that describes the morphology evolution and structural rearrangement that lead to the realizations of highly conductive PEDOT:PSS films and stretchable PEDOT:PSS film with high conductivity, as shown in Figure 1c. The amorphous configuration with large‐domain PEDOT:PSS grains/aggregates (left) is transformed into the highly conductive one with extended chains comprising ordered PEDOT molecules and less PSS (middle). Note that the ordered PEDOT molecules with a variety of reduced π–π stacking distance (*d*) were ionically bonded with PSS molecules. The amorphous configuration of the pristine films can also be transformed into the highly conductive and stretchable ones with an expanded volume and coiled chains that consisted of π–π stacked PEDOT nanofibrils (right). In terms of film stability, unlike opaque metallic electrodes of aluminium (Al), copper (Cu) and silver (Ag) that suffer from a deterioration in conductivity caused by a spontaneous surface oxidation in air,^111^ the optimal PEDOT:PSS films possess satisfactory electrical stability. For example, the strong acid‐modified films reported in literature^78^ showed an outstanding air stability without noticeable degradation in electrical conductivity under high temperature (≈160 °C), probably due to a large removal of the intrinsically hygroscopic PSS chains from the PEDOT:PSS matrix caused by the high‐temperature sulfuric acid (H_2_SO_4_) treatment. The modified CP films are not vulnerable to oxidation and photodegradation when exposed to the air atmosphere. It thus promotes their future commercial applications via developing the stable CP materials. In light of the striking characteristics and behaviors such as a variety of insulator–semiconductor–conductor with a broad range of σ (10^−3^–10^3^ S cm^−1^), high optical transparency (*T*) of >90.0% in visible light range, mechanical compliance, good film‐forming ability, and satisfactory physical and chemical stability against oxidation, thermal and humid conditions,^78^, ^112^ the modified films are suitable to PVs/OLEDs as transparent electrodes,^113^ transistors as interconnectors^109^ or electroactive layers,^114^ and strain gauges as motion‐sensing conductors.^115^, ^116^

**Figure 2:**
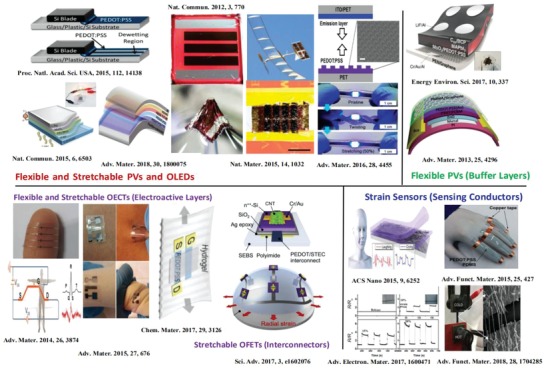
Illustration of the developmental milestone of the flexible and stretchable devices based on PEDOT:PSS from 2012 to 2018. This image gives the device configuration, interface layers, and electrodes for the unprecedented devices. All images reproduced with permission. From ref. 93, Copyright 2015, National Academy of Sciences; from ref. 19, 31, 165, Copyright 2012, 2015, and 2015, respectively, Nature Publishing Group; from ref. 38, 48, 53, 60, 64, 110, 131, 151, Copyright 2015, 2018, 2014, 2017, 2013, 2016, 2018, 2015, respectively, Wiley‐VCH; from ref. 178, Copyright 2017, Royal Society of Chemistry; from ref. 116, 243, Copyright 2015, 2017, respectively, American Chemical Society; and from ref. 109, Copyright 2017, American Association for the Advancement of Science.

This review starts from presenting the fundamental basis of the conduction mechanism of PEDOT:PSS films. Then, it provides a broad overview of the recent progresses in the preparation of highly conductive, flexible, and stretchable PEDOT:PSS films and their bright applications in wearable, flexible, and stretchable electronics such as OSCs, PSCs, OTFTs and strain sensors (**Figure** **2**). The work first demonstrates a wide variety of the CP modification methods that substantially boost the electrical conductivity. Bench mark research results over the past decade are briefly presented in Section 2. In Section 3, methods and strategies to enhancing the strains/elongations of the films are introduced. The sections are divided into three major parts for a clear presentation. In Section 4, we mainly focus on the substantial applications of the modified PEDOT:PSS electrodes in both flexible PVs (i.e., OSCs and OPVs). Section 5 shows the implementations of stretchable PEDOT:PSS films into stretchable electronics including three more specialized categories: strain sensors, PVs, and OTFTs. The PEDOT:PSS films can serve as motion‐sensing conductors for strain sensors, stretchable transparent electrodes for PVs, and interconnectors/electroactive layers for OTFTs, respectively. Finally, challenges and outlooks on the material and device developments are provided at the end of the review. The readers are directed to other excellent review articles^10^ regarding the conductive film preparation and its applications into energy conversion devices.

## 2 Fundamental Considerations for Conductive PEDOT:PSS Films

An ideal flexible transparent electrode (FTE) requires a high electrical conductivity along with mechanical robustness. To describe the electrical conductivity of the PEDOT:PSS films, the sheet resistance (*R*_sh_) of the films is employed. *R*_sh_ and σ can be calculated via van der Pauw method with the following formulas:

(1) $$R_{\mathrm{sh}}=\frac{\pi }{\mathrm{ln2}}R$$

(2) $$\sigma =\frac{1}{R_{\mathrm{sh}}t}$$

Where, *t* is the thickness of the films. The electrical conductivity of PEDOT:PSS electrodes depends on phase separated morphology, PEDOT crystallization, the removal of insulating PSS, PEDOT oxidation, etc. Here, several approaches to boosting the film conductivity are summarized, and the relative mechanisms of conductivity enhancement are demonstrated as well.

### 2.1 Organic Solvents Doping Treatments

In 2002, Zhang and Olle Inganäs reported a PEDOT:PSS anode as indium tin oxide (ITO) alternatives using dropping d‐sorbitl and glycerol into PEDOT:PSS solutions (Baytron P, Bayer AG).^117^ The organic solvent doping is an efficient way of improving the conductivity to two orders of magnitude (from 1 to over 100 S cm^−1^). The underlying mechanism of the conductivity enhancement was unclear yet. Kim et al. used the polar solvent of dimethyl sulfoxide (DMSO) to increase the conductivity of PEDOT:PSS films (Baytron P).^118^ A screening effect was induced by the polar solvent, enabling an improved conductivity up to 80 S cm^−1^. Besides the enhanced conductivity, the DMSO doping endowed the films with a high cohesion among molecules.^119^ It thus benefits to the preparation of the polymeric films with enhanced mechanical reliability. Lee et al.^119^ found that both cohesion and electrical conductivity were improved simultaneously by adding 3 wt% DMSO. The maximum enhancements in cohesion and electrical conductivity were observed where the value was increased by 470% and 6050%, respectively, due to the inter‐PEDOT bridging mechanism. Since then, other doping methods were subsequently developed to obtain a high σ through doping ethylene glycol (EG), glycerol, d‐sorbitol, methanol, X‐triton, and Zonyl fluorosurfactant into PEDOT:PSS aqueous solutions (see **Table** **2**). For instance, Ouyang et al. reported a conductive transparent PEDOT:PSS film, the conductivity of which was improved by two orders of magnitude to 150 S cm^−1^ through optimizing the EG doping contents into PEDOT:PSS aqueous solutions (Baytron P).^120^ With the EG modification, linear and expanded coiled conformations of PEDOT chains were dominated in the as‐modified films, resulting in high conductivity. Despite state of the art progresses on the film conductivity, all those values of conductivity reported still lag far behind that (6740^73^ and ≈13 000^121^ S cm^−1^) of conventional ITO electrodes sputtered on glass substrates.

**Table 2:** Monumental progresses of optoelectrical properties of PEDOT:PSS films

Solvent	Method	Product	*R*_sh_ [Ω sq^−1^]	*T* [%]	σ [S cm^−1^]	Referernce
EG	Doping	PH500	243	89	443	16
	Doping	Baytron P	–	–	200	19
DMSO	Doping	Baytron PH500	190	95	550	73
	Doping	PH1000	191	97	670	18
EG	Shearing	PH1000	16	97.2	4600 (best)	93
EG	Doping and post‐treatments	PH1000	60	86	1330	125
EG	Doping and O_2_ plasma	PH1000	36	73	5012 (best)	186
EG:DMSO	Doping and post‐treatment	PH1000	73	85	–	186
H_2_SO_4_	Soaking (1 m)	PH1000	39	81	3065	78
	Soaking (1 m)	PH1000	67	87	3065	78
	Soaking (100%)	PH1000	46	90	4200	79
	Soaking (98%)	PH1000	79	92	3210	81
H_2_SO_4_	Transfer‐printing	PH1000	45	90	4000	80
	Transfer‐printing	PH1000	33	86.5	4000	80
HNO_3_	Soaking	PH1000	14	–	4100	128
MSA	Soaking	PH1000	–	–	3300	133
MSA	Soaking	PH1000	50	92	2540	187
EG, MSA	Doping, soaking	PH1000	43	92.5	3560	125
Ionic liquids	Doping	PH 1000	31	96	2084	127
	Doping	Baytron P‐V4	–	–	130	126
	Doping	PH1000	59	96	3100	109

### 2.2 Post‐Treatments Combined with Polar Solvent Doping

Apart from adding organic solvents into the PEDOT:PSS aqueous solutions, Ouyang et al.^122^ observed a significant enhancement in film conductivity through immersing PEDOT:PSS (Baytron P) films into EG baths. The film conductivity was enhanced from 0.4 to 200 S cm^−1^ with the polar solvent doping followed by a critical post‐treatment, mostly due to an increased interchain interaction among the PEDOT‐rich domains and a structural change of PEDOT from coil conformation to linear/expanded‐coil conformation. Sankir et al.^123^ also found that the conductivity of PEDOT:PSS (Clevios P) films was enhanced largely via EG post‐treatments. Through inkjet printing followed by the EG post‐treatment, the PEDOT:PSS films coated on flexible underlying substrates exhibited a dramatic improvement in conductivity by 350−900 folds.

**Figure 3:**
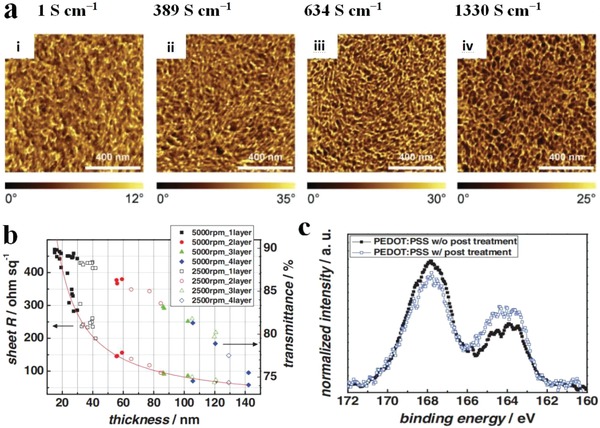
a) AFM images of PEDOT:PSS films: i) Pristine PEDOT:PSS (PH1000) (σ < 1 S cm^−1^), ii) PEDOT:PSS (PH510) with 5 vol% DMSO doping (389 S cm^−1^), iii) PEDOT:PSS (PH1000) with 6 vol% EG doping (634 S cm^−1^), and iv) solvent post‐treated PEDOT:PSS (PH1000) with 6 vol% EG doping (1330 S cm^−1^). b) Metrics of transmittance and *R*_sh_ for the PEDOT:PSS films with solvent post‐treatments versus film thickness. c) XPS S2p spectra of the PEDOT:PSS films. Reproduced with permission.^124^ Copyright 2011, Wiley‐VCH.

The simple post‐treatments are suitable to make conductive PEDOT:PSS films (Clevios PH510 and PH1000). Kim et al.^124^ prepared a transparent PEDOT:PSS film with a conductivity up to 1418 S cm^−1^ by 6 vol% EG post‐treatments. A clear evolution of structural configuration was revealed in phase separation images for the improved conductance (**Figure** **3**a). The pristine PEDOT:PSS films exhibited a typical phase where bright and dark phase represented PEDOT‐rich and PSS‐rich domains, respectively. The pristine films comprising PEDOT‐rich grains had a discontinuous conducting pathway, suggesting an inferior phase separation; whereas the doped PEDOT:PSS films (Clevios PH510 with 5 vol% DMSO, and Clevios PH1000 with 6 vol% EG) had a moderate conductivity of 389 and 634 S cm^−1^, respectively, due to the formation of the favorable phase‐separated morphology that consisted of physically interconnected PEDOT‐rich grains. With the subsequent post‐solvent treatment, the CP films showed a satisfactory conductivity as high as 1330 S cm^−1^, which was attributed to the structural transformation of PEDOT‐rich grains from curved domains to prolonged networks as well as the depletion of insulating PSS. According to the X‐ray photoelectron spectra (XPS) in Figure 3c, the surface ratio of PSS to PEDOT for the as‐cast films and the films with post‐solvent treatments was 2.15:1 and 1.37:1, respectively, indicating that PSS content was remarkably reduced by 36%. We reckon that the large removal of insulating PSS is probably related to the unique immersing of PEDOT:PSS into EG bath, that was, the films were thermally annealed and then immersed quickly into the EG bath. The water‐soluble PSS/PSSH insulators are prone to removal from PEDOT:PSS matrices by washing. Fan et al.^125^ reported a highly conductive PEDOT:PSS film (Clevios PH1000) with the 6 vol% methanol doping treatment followed by a post‐methanol treatment. The PEDOT:PSS films coated on glass substrates showed a further enhancement in conductivity to 2050 S cm^−1^, which was attributed to the formation of more refine PEDOT networks distributed well in the polymeric matrix. Atomic force microscopy (AFM) results demonstrated that the as‐cast PEDOT:PSS films presented an ambiguous phase‐segregated morphology with the root‐mean‐square (RMS) roughness of 5.809 nm; whereas the optimal films with both methanol treatments showed well‐distributed PEDOT networks with RMS of 4.515 nm. Therefore, the combined recipes of doping and post‐treatments are superior strategies than a single doping treatment or a single post‐treatment in terms of film conductivity.

### 2.3 Ionic Liquid Treatments

**Figure 4:**
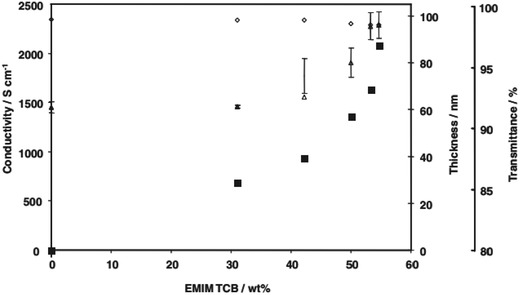
Profiles of conductivity (■), film thickness (△), and transmittance (◊) versus the doping concentration of EMIM:TCB. Reproduced with permission.^127^ Copyright 2012, Wiley‐VCH.

Ionic liquids (ILs) are organic/inorganic salts with striking properties such as good chemical stability, low flammability, and negligible vapor pressure. The dopants have been used to modify PEDOT:PSS films for making the unprecedented optoelectronics.^109^ Dӧbbelin et al.^126^ employed the IL of 1‐butyl‐3‐methylimi‐dazolium tetrafluoroborate ((BMIm)BF_4_) as additives (1.5 wt% in solutions) to increase the conductivity of PEDOT:PSS (Baytron P, H. C. Starck) up to 130 S cm^−1^. The IL swelled the PSS chains and it induced a favorable phase separation with a dimension of about 40−60 nm. A 3D conductive network of conducting PEDOT with small PEDOT grains was formed, leading to an enhanced conductivity.^126^ Badre et al. demonstrated a highly conductive PEDOT:PSS film through doping 1‐ethyl‐3‐methylimidazolium tetracyanoborate (EMIM:TCB, 1.57 wt% in solutions) into PEDOT:PSS aqueous solutions (Clevios PH1000).^127^ With incorporating EMIM:TCB into the CP films, the conductivity was increased dramatically up to 2084 S cm^−1^ (**Figure** **4**). It presented that the PEDOT domains became physically interconnected and larger with the IL modification, thereby resulting in a physically continuous conductive pathway. Through adding IL to PH1000 solutions, it also augmented the ordering of PEDOT segments. Thus, the IL‐modified films exhibited the high electrical characteristic, suggesting a potential application in flexible optoelectronic devices as transparent electrodes. The underlying mechanism of the conductivity enhancement by ILs deserves to be sought further.

### 2.4 Strong Acid Soaking Treatments

A strong acid treatment is considered to be the most effective way to date that substantially boosts the film conductivity by three orders of magnitude (from 1 to over 3000 S cm^−1^).^78^, ^79^, ^80^, ^81^ The innovative recipe is to soak the as‐cast PEDOT:PSS films using highly concentrated strong acids or employing diluted acid aqueous solutions at high temperatures (commonly over 120 °C). In the processing of strong acid modification, H^+^ easily ionized from the acids and it combined the PSS^−^ to form neutral PSSH. The reaction is shown as follows:

(3) $$H^{+}+{\mathrm{PSS}}^{-}\to \mathrm{PSSH}$$

**Figure 5:**
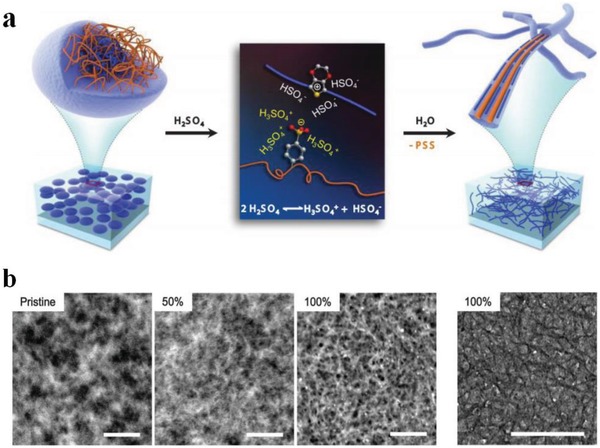
a) Schematic diagram of the structural rearrangement of PEDOT:PSS. The amorphous PEDOT:PSS grains are transformed into highly conducive PEDOT:PSS nanofibrils with 100% H_2_SO_4_ modifications. b) Scanning transmission electron microscope images of PEDOT:PSS films with the H_2_SO_4_ modification at 0, 50, and 100% concentrations, and TEM image of the 100% H_2_SO_4_‐treated film. Scale bars: 200 nm. Reproduced with permission.^79^ Copyright 2014, Wiley‐VCH.

**Figure 6:**
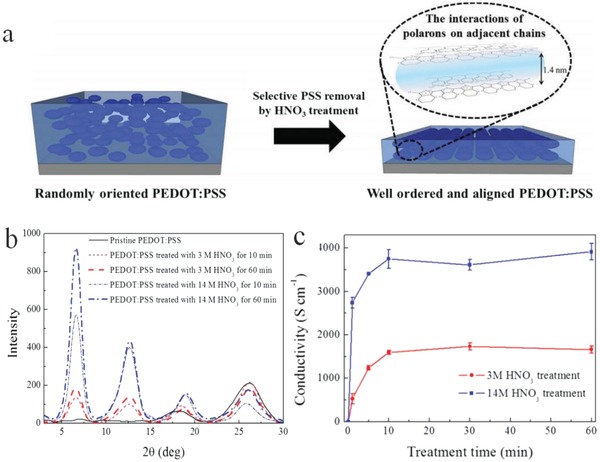
a) Schematic diagram of the structural rearrangement of the PEDOT:PSS films by the HNO_3_ modification. b) XRD patterns of the CP films with and without HNO_3_ treatment. c) Metrics of film conductivity and thickness as a function of the time of HNO_3_ processing. Reproduced with permission.^128^ Copyright 2015, Wiley‐VCH.

Columbic attraction between PEDOT and PSS thus disappeared, and a favorable phase‐segregated morphology was enabled. Moreover, the insulating PSSH chains were segregated from the PEDOT:PSS matrix and they were prone to removal by a deionized water washing.^81^ Because of the favorable phase separation with less insulating PSS and more PEDOT crystalline, the CP films tend to exhibit high conductivity via strong acid modifications.^79^, ^80^ For instance, 1.0 m 78 and 100%^79^ H_2_SO_4_ treatments had been employed to modify the CP films. The harsh strong acid treatments induced a record‐high conductivity (3065^78^ and 4380 S cm^−1^ 79) and high transparency with a plateau over 90% in the visible region required by high‐merit transparent electrodes. Lee et al.^79^ observed that the pristine films consisted of many agglomerates (white contrast) in a granular shape; whereas with 50 wt% H_2_SO_4_ treatments, the films showed dilatational grains (**Figure** **5**). As the H_2_SO_4_ concentration was increased to 100%, a drastic transformation occurs in the morphology from a granular shape to PEDOT‐rich nanofibrils with a width of 10–15 nm. The results indicate that the strongly acidic H_2_SO_4_ treatment leads to a structure restructuring for the PEDOT:PSS films, that is, the favorable networks comprising the crystallized PEDOT‐rich nanofibrils. Yeon et al.^128^ demonstrated a method of 67 wt% nitric acid (HNO_3_) treatments at room temperature that enabled the thin film (≈30 nm) with outstanding conductivity up to 4100 S cm^−1^. The HNO_3_ treatment induced a better phase separation and more refined nanofibrils through selectively removing the PSS components (**Figure** **6**). Amorphous PEDOT:PSS grains are reformed to crystalline PEDOT:PSS structures. This structural rearrangement of PEDOT substantially accounted for the best conductivity up to 4100 S cm^−1^ through the enhancement of the interchain interaction in the films. Also, Sun et al.^129^ reported a conductive PEDOT:PSS film with a value of 1100 S cm^−1^ through addition of chloroplatinic acid (H_2_PtCl_6_). The underlying mechanism of conductivity enhancement is mainly attributed to phase separation of PEDOT:PSS. Because PEDOT was doped and oxidized by Pt^4+^ of H_2_PtCl_6_ (Pt^4+^ is reduced simultaneously to Pt^2+^), proton transfer was allowed from the acids to PSS, causing the formation of neutral PSSH.^129^ A favorable phase separation with conformation changes of PEDOT chains thus occurred in the films. Additionally, the H_2_PtCl_6_‐doped PEDOT:PSS films exhibited 84% transmittance, good air stability, and superior robustness in bending tests.

**Figure 7:**
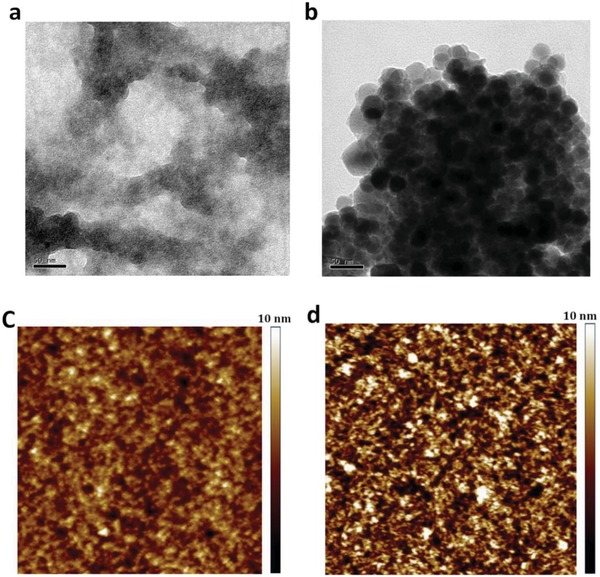
TEM and AFM images of pristine films and the films with the gentle acid treatments at room temperature. a,c) Pristine films. b,d) Films with the gentle acid treatments. Scale bar: 2 × 2 µm^2^. Reproduced with permission.^131^ Copyright 2018, Wiley‐VCH.

However, the CP films coated on hydrophobic flexible plastic substrates are inferior to the ones coated on rigid glass substrates in terms of conductivity, uniformity, and smoothness. It is challenging to directly prepare highly conductive PEDOT:PSS films on flexible plastic substrates,^79^, ^80^, ^81^, ^125^, ^130^, ^131^ mostly due to the destructive reactions between strong acids and plastic underlying substrates that is detrimental to the plastic substrates used. To solve the key issue, a feasible method is to employ gentle acids (i.e., methanesulfonic acid [MSA],^81^ oxalic acids and phosphoric acids^132^) instead of harsh H_2_SO_4_. Using the gentle acid modifications, the PEDOT:PSS films coated on plastic substrates can exhibit a high conductivity of 1500−3000 S cm^−1^ without visible mechanical damages. For instance, Fan et al.^131^ developed the method of 99 wt% MSA soaking treatments at room temperature that avoided the formation of large‐domain aggregates and restrained the damage of strong acids to polyethylene terephthalate (PET) substrates. As shown in **Figure** **7**a, no obvious aggregates of PEDOT:PSS were found in the pristine films. After the gentle acid treatments, a number of small aggregates appeared and were physically connected well (see Figure 7b), suggesting a better phase‐separated morphology. Notably, the aggregates became physically continuous and consisted of smaller particles with sizes of 20–40 nm as compared to that (≈100 nm) of PEDOT:PSS aggregates with high‐temperature MSA modifications.^133^ AFM showed that the pristine film had a poor phase‐separated morphology along with an RMS of 1.67 nm.^131^ With the gentle acid treatments, the film had smooth surfaces with an RMS of 2.14 nm and it showed refine PEDOT‐rich nanofibrils. Besides, the work function (WF: −4.91 eV) of the CP film with the MSA treatment at room temperature matched well with the WF (−5.05 eV) of PEDOT:PSS (Clevios P VP AI4083) buffer layers. It is energetically favored for hole transport and extraction by the CP electrodes. Notably, the gentle acid treatments restrained the detrimental reactions that often occurred between harsh strong acids (e.g., H_2_SO_4_ and HNO_3_) and underlying plastic substrates. Besides the commonly used acid socking treatments, an acid predoping treatment (that is adding acids into PEDOT:PSS aqueous solutions) was developed.[qv: 49a,129] The unique acid treatment has the advantages of simple manufacturing and compatibility with various printing techniques for mass production.

### 2.5 Acid‐Assisted Transfer‐Printing Strategies

**Figure 8:**
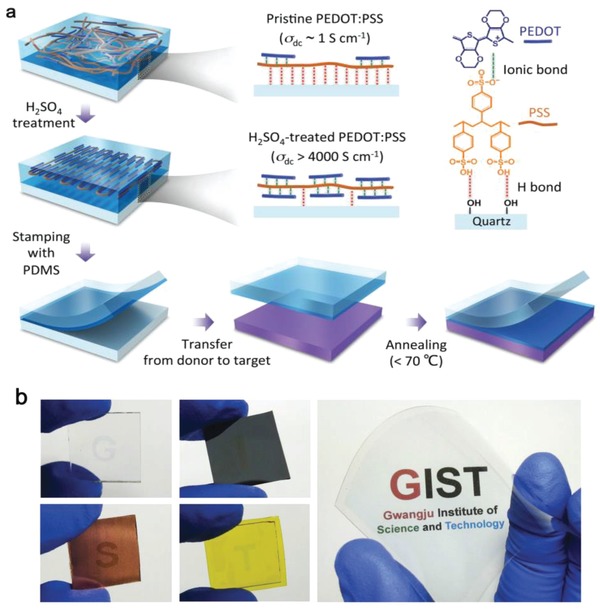
a) Schematic diagram of transfer‐printing processing. b) Photographs of PEDOT:PSS films that were transferred onto various substrates, including glass slides, silicon wafers, Cu foils, kapton tapes, and large‐size PET foils marked with GIST logos. Reproduced with permission.^80^ Copyright 2015, Wiley‐VCH.

Most strong acid modifications do induce a high conductivity for the PEDOT:PSS films, but these strong acids are rather harsh and strongly corrosive to the plastic substrates especially under high‐temperature curing. It commonly caused a large‐domain damage to the underlying plastics with irregular configurations as well as environment and safety concerns. To solve the issues, Kim et al.^80^ reported a unique H_2_SO_4_‐assisted transfer‐printing strategy that avoided the direct contacts between strong acids and plastic substrates (**Figure** **8**). It enabled the flexible polymeric films with a record‐high conductivity of 4100 S cm^−1^.^29^ However, the H_2_SO_4_‐assisted transfer‐printing technique suffered from several drawbacks such as safety and environment concerns and strong acid residues that blocks its applications. Fan et al.^81^ developed a mild acid (MSA)–assisted transferring method that not only weakened the adhesion between PEDOT:PSS and quartz substrates but also increased the adhesion between PEDOT:PSS and the target objects of poly(dimethylsiloxane) (PDMS) stamps. Thus, it enabled the highly conductive CP films to be transferred onto PDMS. The transferred films exhibited a high conductivity of 3500 S cm^−1^, which was comparable to that (3200 S cm^−1^) of the transferred ones with H_2_SO_4_ modification. Notably, the transferred films with MSA modifications are superior to the later ones with H_2_SO_4_ modifications in terms of mechanical compliance and optical transparency, mostly due to the usage of the mild acid modification with less acid residues. However, the transfer‐printing (or transferring) methods mentioned above required a precise adjustment of van der Waals (VDW) interactions at interfaces, resulting in a lack of reproducibility of the CP products. Besides, the methods consisted of more complex operation procedures and tended to cause a low yield of large‐area films,^60^, ^132^ thereby minimizing the advantages of these unique methods. Recently, a highly viscous acid‐free dipping‐embedded‐transfer method was reported to significantly improve the yield of the PEDOT:PSS films with large size.^60^ It enabled a highly conductive all‐plastic electrode with a structure of PEDOT:PSS embedded into PDMS elastomers (namely PEDOT:PSS−PDMS). The robust adhesion between PEDOT:PSS and target objects of PDMS avoided the key issues such as low yield and small size for the transferred or transfer‐printed films. Notably, the resulting films bear substantial advantages including high yield of approaching 100%, large area of at least 4.7 × 4.7 cm^2^, high flexibility and good electrical stability.^60^ In terms of optical and electrical characteristics, the transferred films yielded a high conductivity of 2890 S cm^−1^ with a transparency of 95% at λ = 550 nm. It is envisioned that efficient flexible optoelectronics with the high‐performance CP electrodes have a high probability of being realized.

The electrical conductivity of the flexible PEDOT:PSS films remains far behind that (6740^73^ and ≈13 000^121^ S cm^−1^) of rigid ITO films on glass substrates and metallic films (e.g., Ag: 6.3 × 10^7^ S cm^−1^ and Al: 3.5 × 10^7^ S cm^−1^). For their practical implementations into modules, new strategies to making a desirable polymeric electrode with striking characteristics (i.e., *σ* > 6000 S cm^−1^, *T* > 95%, and high flexibility without sacrificing its conductivity in harsh flexing at a bending radius (*r*) of ≤1.0 mm) are needed.

## 3 Mechanical Properties: Toward Robust Stretchable PEDOT:PSS Films

For the unprecedented applications of wearable and stretchable electronics, it is critical to develop the stretchable conductive films/conductors that can retain most of initial electrical conductivity under mechanical insults. Although the PEDOT:PSS films are regarded as a promising candidate of flexible and transparent electrodes,^60^, ^78^, ^79^, ^80^, ^81^, ^125^ the intrinsic stretchability of as‐cast PEDOT:PSS films is only ≈2% elongation at break, rendering them unsuitable to stretchable electronics. Developing the intrinsically stretchable materials (at least affording 20% elongation) has become urgent and important. So far, the reported stretchable CP films have been fabricated through three main routes: geometric/strain engineering, elastomeric blends, and doping modification.^134^, ^135^, ^136^, ^137^, ^138^ **Table** **3** summarizes the stretchable property along with the electrical characteristics of the PEDOT:PSS stretchable films/conductors reported.

**Table 3:** Brief summary of current PEDOT:PSS stretchable films/conductors

Treatment	Film	Processing	Electrical characteristics	Mechanical test	Reference
Prestraining	PH1000	15% prestraining	150 Ω, no changes in *R* under ε: 0.1	5 × 10^3^ cycling	139
	PH1000	57% prestraining with EMIM:TCB doping	σ > 1000 S cm^−1^, no changes in *R* under ε: 1.8	Cycling	154
Elastomer blends	PU:PH1000 blends	Mixed with PU ITF solutions	100–110 S cm^−1^ in straining at ε: 1.0	Cycling	142
	PU:PH1000 blends	Mixed with PU ITF solutions	From 100 to 35 S cm^−1^ with ε: 2.0	Cycling	142
	PDMS:PH1000 blends	PDMS blending	2–3 Ω sq^−1^ in straining at ε: 0.1	5 × 10^3^ cycling	150
DMSO/Zonyl doping	PH1000	–	470 KΩ sq^−1^, no changes in *R* under ε: 0.2	20 cycling	151
Ionic liquids	PH1000	STEC enhancer doping	3000–4000 S cm^−1^ in straining at ε: 1.0	10^3^ cycling	109
	PH1000	STEC enhancer doping	From 3000 to 600 S cm^−1^ with ε: 6.0	10^3^ cycling	109
	PH1000	EMIM:TCB doping	>1000 S cm^−1^ with ε: 0.2 (relaxing state)	Cycling	153
Soft materials	PVA:PH1000 blends	PVA89K blending	75 S cm^−1^ with ε: 0.5	Loading	152
	PVA:PH1000 blends	PVA89K/EG blending	172 S cm^−1^ with ε: 0.5	Loading	152
	Acid‐PH1000/PVA:PH1000	Acid and PVA treatments	3000 S cm^−1^ with ε: 0.2	400 cycling	210
Acid‐assisted transferring	PH1000	Embedding into PDMS	3000 S cm^−1^ with ε: 0.3	300 cycling and loading	60

### 3.1 Geometric/Strain Engineering

In the first approach, the conducting materials are geometrically coated or patterned into wavy lines that can be extended when the supporting elastomeric substrate is stretched. Through the advisement of intelligent geometrical structures, stretchable lines with a geometrical configuration are prone to obtainment; whereas in the case of stretchable film fabrication, a thin film of conductive materials was often deposited on a prestrained elastomeric substrate, leading to the formation of periodic buckles/wrinkles upon releasing the strained substrates. It enabled a buckled/wrinkled structure that had ability to afford repeated stretching–releasing. For instance, Bao et al.^139^ prepared highly conductive and stretchable PEDOT:PSS films coated on PDMS stamps that were prestrained to 15%. Buckled structures were formed when the elastomeric PDMS substrate was relaxed. The PEDOT:PSS/PDMS samples were able to withstand the 10% strain of 5000 stretching–releasing cycles without obvious changes in conductance. Four‐layer PEDOT:PSS films had a *R*_sh_ as low as 46 Ω sq^−1^ with 82% transmittance (at 550 nm). Seol et al. reported a stretchable composite film that consisted of PEDOT:PSS and reduced graphene oxide (r‐GO).^140^ The PEDOT:PSS/r‐GO composites were coated on a prestrained PDMS, exhibiting a good stability in stretching–releasing tests at 15% strain. The prestraining strategy has a high possibility of transforming virtually any rigid materials to stretchable materials while retaining most of the initial electrical conductivity. In light of the strategy, the prestrain‐dependence stretchability of over 200% strain will be obtained probably for the polymeric films. However, the formation of buckled/wrinkled structures blocks the CP materials integration into flexible and stretchable electronics that commonly require planar configurations or low profiles. In summary, these strategies endow the devices with outstanding stretchable properties but do not seem to be suitable to the development of flexible and intrinsically stretchable devices.

### 3.2 Elastomeric Blends

The second major route used to make stretchable conductors is to embed conductive fillers into an insulating elastomeric matrix, leading to the formation of nanocomposites.^141^, ^142^ Typically, PEDOT:PSS, CNTs, and Ag NWs have been used as the conductive fillers.^142^, ^143^, ^144^, ^145^, ^146^ However, the percolation‐dependent conductivity of the candidates is rather sensitive to tensile strains, because of a high probability of being broken with crack propagation under large strains of over 20% for the polymeric and metallic films.^147^, ^148^, ^149^ It thus affected the device durability and stability. Numerous researches have been devoted to the realization of durable stretchable conductors with good stability. For instance, Larsen et al.^142^ demonstrated a highly stretchable conductive CP material through blending the PEDOT:p‐tosylate and an aliphatic polyurethane (PU) elastomer. The blends showed a moderate conductivity of 120 S cm^−1^ and had an elastomeric mechanical property closely resembling that of the PU. By stretching the blend material to 50% strain, it led to an increase in conductivity; while subsequently relaxing the blends, the conductivity was decreased. Thus, the blends may be used to fabricate soft strain sensors that detect the motions of targets. Meaningfully, the blend system is a single phase and not two separate phases.

Teng et al.^150^ prepared a highly stretchable PEDOT:PSS@PDMS composite conductor. The 3D elastic network formed by in situ polymerization serves as a support for the 3D network of the brittle PEDOT:PSS aerogel. The as‐prepared PEDOT:PSS@PDMS conductor retained a high elasticity up to 43% strain, and it could maintain the electrical stability over a number of tensile stress cycles at 10% strain. Despite the enhanced stretchability of the films with 3D network, the doping of insulating materials such as PDMS or PU failed to evolve the film phase separation and to remove the insulating PSS from the matrices, therefore, it is challenging to make highly conductive polymer conductors. Indeed, using the second method of composite strategies, the stretchable PEDOT:PSS films commonly suffered from a limited conductivity of no more than 100 S cm^−1^.

### 3.3 Plasticizer, Soft Material, and IL Blending

In general, high crystallinity and less insulating components/fillers are definitely favorable for satisfying the high conductivity required by the PEDOT:PSS electrodes. For making intrinsically stretchable polymeric films with high conductivity, the PEDOT:PSS films should be swelled, that is, a large free volume between coiled PEDOT and PSS chains is needed to make the films amenable to mechanical insults and tensile strains. As such, the last strategy to make the stretchable CP conductors is proposed via the treatments of plasticizers, soft materials, ILs, etc.

**Figure 9:**
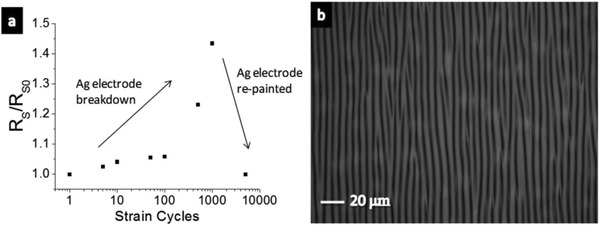
a) Normalized sheet resistance versus stretching cycles with 10% strain for the buckled PEDOT:PSS film with 1 wt% Zonyl. b) Optical microscopy image of the buckled PEDOT:PSS film (0% strain). Reproduced with permission.^139^ Copyright 2012, Wiley‐VCH.

**Figure 10:**
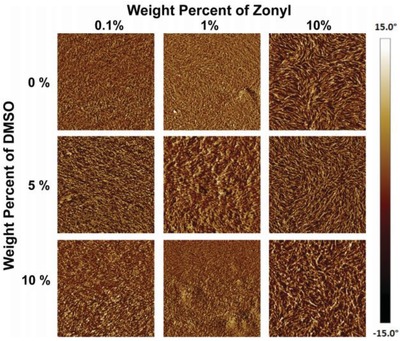
AFM phase images of PEDOT:PSS films as a function of the content of DMSO and Zonyl FS‐300 in the solution used for deposition. The dimensions of each AFM image are 1.5 µm × 1.5 µm. Reproduced with permission.^151^ Copyright 2012, Wiley‐VCH.

The stretchable PEDOT:PSS films were enabled by incorporating plasticizers such as Zonyl^151^ and Triton.^110^ Note that the Zonyl plays three roles including: i) plasticization of the CP for improving the stretchability; ii) improving the wetting property of PEDOT:PSS aqueous solutions for intimate contacts; and iii) boosting the electrical conductivity of the CP. For instance, Bao et al.^139^ presented a stretchable and conductive PEDOT:PSS (Clevios PH1000) film by adding 5 wt% DMSO and 1 wt% Zonyl FS‐300. As shown in **Figure** **9**, the stretchable films coated on prestrained PDMS substrates were able to withstand over 5000 stretching cycles of 10% strain with no change in resistance (*R*_sh_ of 240 Ω sq^−1^ with 97% transparency at λ = 550 nm). Because of the hygroscopic nature of PSS and the plasticization, the PEDOT:PSS films were swelled and became relatively soft as compared to the rigid conjugated polymer of PEDOT. The electrical conductivity was improved as well, which was attributed to the favorable phase separation that consisted of elongated grains/aggregates and physically continuous conductive pathways. However, the films treated by 10 wt% Zonyl had a relatively low transparency, rendering them unsuitable to flexible and stretchable PV/display devices. Then, Lipomi et al.^151^ investigated the effects of common additives (i.e., DMSO and Zonyl FS‐300) on the electrical and mechanical behaviors of the CP films. All samples containing high‐concentrated Zonyl exhibited high‐aspect‐ratio fibrils. The samples with 10 wt% Zonyl had the greatest mechanical compliance (**Figure** **10**). Film elasticity was able to maximize to 28% tensile strain because of film plasticization, which was induced by the adding of 10 wt% Zonyl FS‐300 and 5 wt% DMSO. Besides, the films retained a modest conductivity of 150 S cm^−1^ in stretching–relaxing tests.

**Figure 11:**
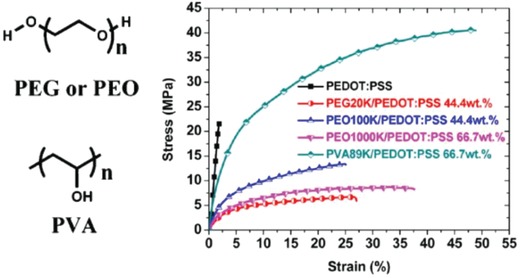
Representative stress–strain characteristics of the neat PEDOT:PSS films and the PEDOT:PSS blends with soft polymers of PEG, PEO, and PVA (see molecule structures in the left). Reproduced with permission.^152^ Copyright 2013, American Chemical Society.

Through blending the PEDOT:PSS solutions with high‐loading soft polymers such as poly(ethylene glycol) (PEG), poly(ethylene oxide) (PEO) and poly(vinyl alcohol) (PVA), the elongation at break may be increased due to the good miscibility of soft polymers and the bonding among soft materials, PEDOT:PSS, and substrates. For example, Li et al.^152^ prepared the stretchable PEDOT:PSS films (thickness ≥ 1 µm) by casting the aqueous solution of PEDOT:PSS (Clevios PH1000 blended with soft polymers). The incorporation of PEG20K, PEO100K, or PEO1000K minimized the tensile strength but increased the elongation at break, while PVA89K can simultaneously increase the tensile strength and the elongation at break. (**Figure** **11**). However, with an increase in the weight fraction of PEO1000K or PVA89K to 75.0 wt%, it caused the formation of a gel that was hardly processed for making free‐standing films. The results show that the film blends showed a satisfactory elongation at break as large as 50% strain and the best conductivity up to 172 S cm^−1^ via the optimal recipe (i.e., 3 vol% EG and 66.7 wt% PVA89K blending). Although the drop‐cast film blends lag far behind the modified PEDOT:PSS films with strong acid modifications in terms of conductivity, the work provides a new strategy to make stretchable and conductive PEDOT:PSS blends that have a potential of applications into wearable and stretchable electronics.

**Figure 12:**
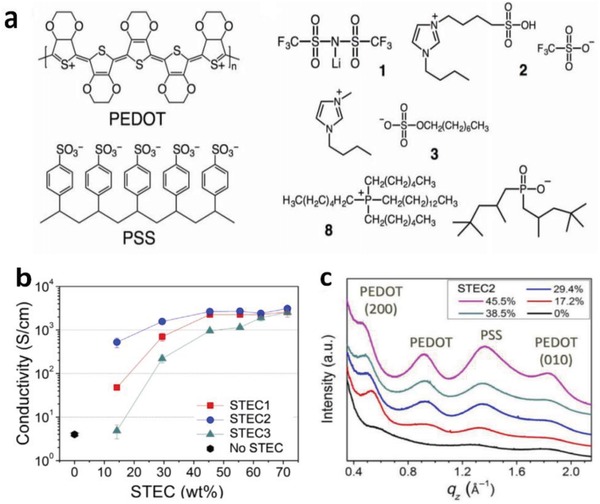
a) Chemical structures of PEDOT:PSS and representative STEC enhancers. b) Conductivity of the PEDOT films. c) Near out‐of‐plane intensity plot of PEDOT:PSS films with various amounts of STEC2 additives. Reproduced with permission.^109^ Copyright 2017, American Association for the Advancement of Science.

Ionic liquid doping is the most commonly used method for the simultaneous obtainment of large stretchability and high conductivity for the PEDOT:PSS films. The IL materials not only act as a secondary dopant for improving the film conductivity, but also serve as a plasticizer for prolonging the film elongations. Monumental progresses had been achieved in the case of the stretchable conductor fabrication.^153^, ^154^, ^155^ For example, Kee et al.^153^ reported the stretchable PEDOT:PSS transparent electrodes through mixing the ionic liquid compound 1‐ethyl‐3‐methylimidazolium:tetracyanoborate (EMIM:TCB, 1.3 wt%). The films with EMIM:TCB modification showed a low *R*_sh_ of 67 Ω sq^−1^ and a high transparency of 91% at the visible‐light range, demonstrating nearly identical optical and electrical characteristics to that of commercial products of ITO/PET substrates. Importantly, the CP electrodes became flexible in the extreme and can afford the harsh mechanical tests at a bending radius down to 0.7 mm. Furthermore, the CP films prepared on PDMS even afforded the tensile strain as large as 30% and showed stable resistances that just increased by 57%. Besides, Teo et al.^155^ reported a highly stretchable and conductive PEDOT:PSS film incorporated by the ionic liquid of EMIM:TCB (1.5 wt% in solutions). It resulted in an enhanced stretchability of 50% strain along with a high conductivity of 1280 S cm^−1^ for the IL‐modified films. The films have a bright promise for making the ubiquitous PV/displays featuring all‐printing, ultra‐flexible, and semitransparent characteristics. Later, a significant breakthrough to making stretchable conductors was made by Bao's group.^109^ The stretchability of CP films was enhanced by a verity of IL enhancers that served the dual functions: i) morphology evolution, and ii) acting as conductivity‐enhancing dopants in PEDOT:PSS films. As shown in **Figure** **12**, the CP films exhibited a high conductivity of over 3100 S cm^−1^ under normal conditions (i.e., 0% strain), and 4100 S cm^−1^ under 100% strain among the highest values for stretchable conductors reported so far. Notably, the CP films were highly durable under harsh conditions of cyclic loading, that is, the films had the ability of retaining a satisfactory conductivity of 3600 S cm^−1^ even in the 1000 cycle stretching–releasing tests at 100% strain. Especially, the film retained a conductivity of beyond 100 S cm^−1^ under the extreme strain as large as 600% and it had a fracture under the strain up to 800%, which are superior to even the best Ag NW‐ and CNT‐based stretchable conductors. The combination of outstanding electrical and mechanical characteristics allows the CP films to serve as stretchable interconnects for making field‐effect transistor arrays with a high device density. However, the large aggregates in PEDOT:PSS solutions perhaps caused uniformity and smoothness concerns in the as‐prepared PEDOT:PSS films (thickness ≥ 600 nm). As a consequence, the uniform, smooth and transparent PEDOT:PSS films having electro‐optical trade‐offs better than state‐of‐the‐art ITO products should be continuously pursued in the future.

## 4 Film Stability

The electrical stability of the PEDOT:PSS film is one of the pivotal considerations for flexible electronics. Unlike the metallic configurations (e.g., films, grids, meshes, nanowires, nanoparticles) that are the most commonly used FTEs, the conductive polymers with modifications can obtain a satisfactory electrical stability in the ambient atmosphere. In addition, the solution‐processing of PEDOT:PSS electrodes is highly desirable for the printing and roll‐to‐roll manufacturing of cost‐efficient flexible productions. Therefore, the stable CP films are considered to be critical to the practical implementations of printed modules.

The as‐cast CP films mainly suffer from poor stability against heat and moisture when exposed to air atmosphere.^156^ Choulis et al.^157^ demonstrated the thermal degradation mechanisms of PEDOT:PSS films (PH500). Thermal aging starts with an increase of the mobility of the hole polarons in PEDOT oligomers attached to insulating PSS chains. Then, the ionic bonds between PEDOT and PSS would be separated with thermal annealing, leading to the formation of aligned PSS chains and PEDOT‐rich aggregates/grains. The morphology evolution resulted in an enhancement in electrical conductivity. Finally, as PEDOT and PSS were breaking, the conductive PEDOT being hydrophobic would concentrate into the interior of the aggregation/grains, while the insulating PSS being hydrophilic would concentrate at the borders of the aggregation/grains, thereby enhancing the potential barriers between the grains. However, the pioneering research focused on the stability of as‐cast films with a poor conductivity of 1.2 S cm^−1^ at 200 K and 2.2 S cm^−1^ at 370 K; whereas for highly conductive PEDOT:PSS films, there were rare reports regarding the heat‐transfer mechanism on film structures and performances. The configuration change of PEDOT aliment/aggregation and PSS chains also deserves to be sought further.

**Figure 13:**
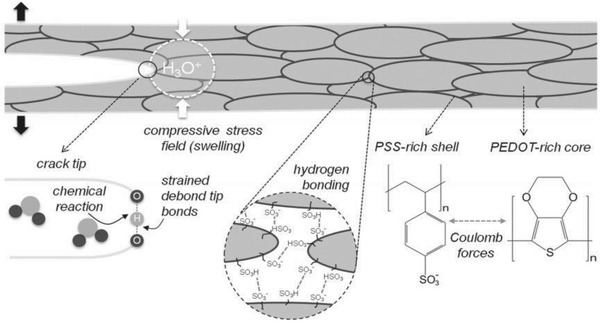
Decohesion mechanism of PEDOT:PSS films. The PEDOT:PSS grains consisted of a PEDOT‐rich core and a PSS‐rich shell that were held together by hydrogen bonds. Due to the absorption of water, it caused a stress‐dependent chemical reaction between water molecules and hydrogen bonds at the debond tip. Reproduced with permission.^158^ Copyright 2014, Wiley‐VCH.

The other concern is that PEDOT:PSS films absorb water from the surrounding humid environment and then become swelled owing to the hygroscopic property of PSS. It is thus detrimental to the structural stability of PEDOT:PSS. Dupont et al.^158^ demonstrated the decohesion kinetics of PEDOT:PSS conductive films under varied environmental conditions with changing relative humidity and temperature. As illustrated in **Figure** **13**, as‐cast PEDOT:PSS films were susceptible to moisture‐assisted decohesion. The individual grains of PEDOT:PSS were held together by hydrogen bonds. During moisture‐assisted decohesion, the bonds at the debond tip were strained, which weakened the hydrogen bond interactions. Water molecules near the debond tip might react with the weak hydrogen bonds through the following chemical reaction 159:

(4) $$H_{2}O+\mathrm{PSS}\;\left({\mathrm{HSO}}_{3}\right)\to H_{3}O^{+}+\mathrm{PSS}{\left({\mathrm{SO}}_{3}\right)}^{-}$$

When the PEDOT:PSS films were under humid environments, debond propagation was caused by a synergistic effect of mechanical stresses and water molecules. Thus, the hydrogen bonding that was predominant in bonding the individual PEDOT:PSS grains accounted for the decohesion mechanism.

**Figure 14:**
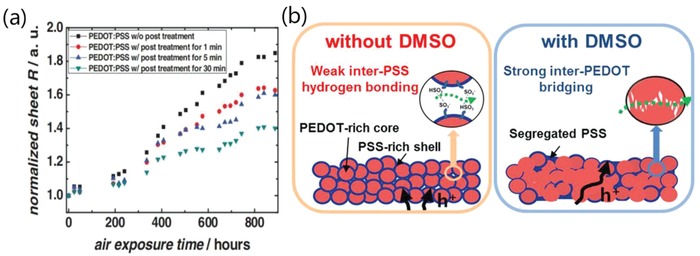
a) Profiles of the *R*_sh_ of PEDOT:PSS films with solvent post‐treatments as a function of air exposure time. Reproduced with permission.^124^ Copyright 2011, Wiley‐VCH. b) Schematic diagram of the debonding mechanism of PEDOT:PSS before and after mixing with DMSO. Reproduced with permission.^119^ Copyright 2016, American Chemical Society.

Two methods are introduced to enhance the stability of the CP films. The first method is the post‐treatment of secondary solvents. Kim et al.^124^ reported that the air exposure did cause a gradual increase in *R*_sh_ for all the EG‐modified CP films, which was attributed to water absorption from the air atmosphere (**Figure** **14**a). The films with the EG post‐treatments showed a lower *R*_sh_, compared to the ones without post‐treatments; the optimal films with the long‐time (≈30 min) post‐treatment showed the best stability against air exposure, indicating that the depletion of PSS minimized the water absorption. The better stability perhaps corrected with the strong cohesion between PEDOT and PSS caused by secondary solvents. It is reported that the cohesion and electrical conductivity were significantly affected depending on the weight percent of DMSO.^119^ It is also found that 3 wt% DMSO was the optimum recipe for highly mechanical and electrical properties. After the optimum DMSO doping, the maximum enhancements in cohesion and conductivity were observed where the values were increased by 470% and 6050%, respectively, due to the inter‐PEDOT bridging mechanism.

**Figure 15:**
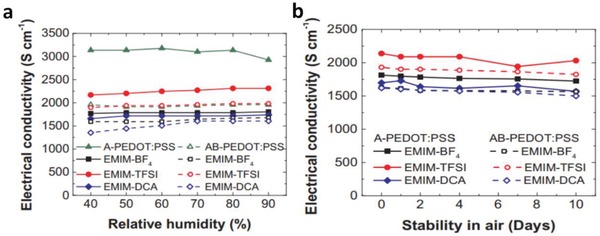
Metrics of electrical conductivity of the PEDOT:PSS films treated by IL versus a) relative humidity and b) storage days. IL concentration in methanol is 25 vol%. A‐PEDOT:PSS is the film with H_2_SO_4_ treatments; and AB‐PEDOT:PSS is the film with H_2_SO_4_ and IL treatments. Reproduced with permission.^112^ Copyright 2018, Elsevier.

**Figure 16:**
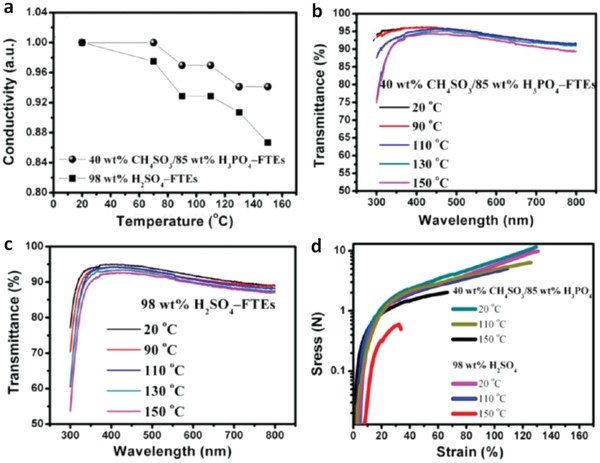
Comparison of a) conductivity, b,c) transmittance, and d) stress−strain curves of the PEDOT:PSS anodes with gentle acid and H_2_SO_4_ treatments. Reproduced with permission.^81^ Copyright 2016, American Chemical Society.

The second method for a better stability is a strong acid socking treatment. Strong acid socking treatments as aforementioned above removed a large amount of PSS/PSSH from the polymeric matrix, thus the acid‐modified films exhibited an enhanced stability. For instance, Ouyang et al. reported that the H_2_SO_4_‐treated PEDOT:PSS film exhibited an outstanding air stability with a consistent conductivity.^78^ The acid‐modified films almost had no obvious change in electrical conductivity after two months. Furthermore, the CP films that underwent H_2_SO_4_ soaking treatments followed by a verity of IL treatments also could retain over 90% of the original conductivity in the air for 10 days,^112^ suggesting a long‐term ambient stability of the H_2_SO_4_‐modified PEDOT:PSS films (**Figure** **15**). The underlying mechanism of the enhanced air stability was not fully indicated yet. Recently, Fan et al. found that a verity of acid treatments, acid residues and heat treatments had a significant effect on the film performances including conductivity, transparency, stability and mechanical flexibility (**Figure** **16**).^81^ XPS results demonstrated a large removal of hygroscopic PSS components from the PEDOT:PSS matrix, which accounted for the high stability of the films under normal air conditions. However, compared with the mild acid‐treated films, the H_2_SO_4_‐treated ones exhibited the lower electrical conductivity and optical transparency, mostly due to the H_2_SO_4_ residues that destroyed the underlying substrates. With increasing the heat temperature of thermal annealing from 20 to 150 °C, the flexible substrates were destroyed visibly, leading to a sharp deterioration in optical transparency and mechanical flexibility of the CP films coated on PDMS.

Mild acid treatments are definitely favored to improve the air stability and the flexibility for the PEDOT:PSS films because largely remove the hygroscopic PSS from PEDOT:PSS matrices and protecting the underlying plastic substrates from harsh processing of strong acids. We advise to employ the compact passivated layers (e.g., graphene and r‐GO) to restrain the humid penetration into the PEDOT:PSS matrices. It is energetically favored for enhancing the air stability of both the CP films and these electronics integrated with the CP.

## 5 Flexible PVs

Flexible PVs that turn light into electricity become emerging as wearable, sustainable, and environmentally friendly energy sources.^160^, ^161^, ^162^, ^163^, ^164^, ^165^ The fabrication of flexible and transparent electrodes plays a key role to govern device performance. However, the traditional electrodes of ITO suffer from a limited conductivity on plastic substrates, and they are susceptible to crack propagation in flexing and under tensile strains,[qv: 9b,11] hampering their applications into flexible PV fields. Numerous efforts had been devoted to the subjects of ITO alternatives such as CPs,^78^, ^79^, ^80^, ^81^ metal NWs,^166^, ^167^ CNTs,^168^, ^169^, ^170^ metal meshes or grids,^171^, ^172^ and hybrid electrodes comprising PEDOT:PSS components.^173^, ^174^, ^175^ Among them, the classic CP (i.e., PEDOT:PSS) has received intense researches, and it has the grand advantages such as solution processability, high optical transparency, cost‐effective fabrication, flexibility, and excellent thermal stability. So far, the highest power conversion efficiency (PCE) of lab‐size ITO‐free single‐junction flexible OSCs and flexible PSCs have been exceeded 10%^176^, ^177^ and 16%,^178^ respectively. Much effort has been devoted to the improvement in PCE, mechanical compliance and stability of the flexible PV devices by the modifications of PEDOT:PSS films.

It is critical to develop the technologies of FTE preparations. They enable the next‐generation devices featuring high flux‐per‐weight (e.g., power‐per‐weight of beyond 20 W g^−1^ for PVs), ultrathin design, and printable manufacturing. Because of the simple solution‐preparation and the striking merits in smoothness and uniformity, PEDOT:PSS films are the most commonly used electrode materials toward stretchable and ultrathin optoelectronic applications. In this section, we highlight historically monumental research results and recent state‐of‐the‐art progresses on flexible OSCs and PSCs with PEDOT:PSS and its hybrid electrodes.

### 5.1 ITO‐Free and Flexible OSCs

#### 5.1.1 OSCs with PEDOT:PSS Electrodes

In 2002, Zhang et al.^117^ used the modified PEDOT:PSS (Baytron P, Bayer AG) as ITO alternatives to fabricate OSCs. The sheet resistance of the PEDOT:PSS electrode was decreased from 1.5 × 10^5^ to 1.3 × 10^3^ and to 1.0 × 10^3^ Ω sq^−1^ by the the doping treatments of glycerol and sorbitol, respectively. The resulting ITO‐free OSC devices exhibited a PCE of ≈3.0%. Although it lags far behind that (≈5.4%) of the ITO‐based OSCs, the historically monumental research gave rise to intense studies with respect to PEDOT:PSS modification and flexible PV fabrication. Ouyang et al.^120^ reported an efficient ITO‐free OSC with EG‐doped PEDOT:PSS anodes and MEH‐PPV active layers that exhibited an efficiency of 1.5%. A mechanism of enhancement in the anode conductivity was proposed as follows: the organic additive of EG induced a conformation change of PEDOT chains in the PEDOT:PSS matrix from mixed coils to linear/expanded‐coils, thereby resulting in an increase of charge‐carrier mobility. In 2008, Na et al.^73^ reported highly efficient ITO‐free OSCs with PEDOT:PSS anodes (Clevios PH500 doped with 5% DMSO) fabricated on glass and PET substrates, respectively. It is found that the DMSO‐doped PEDOT:PSS films recorded a peak conductivity of 550 S cm^−1^ with an average value of 470 S cm^−1^, but the conductivity still lagged far behind 1050 S cm^−1^ of ITO coated on PET substrates and 6740 S cm^−1^ of ITO coated on glass substrates,^73^ respectively. In addition, upon a mechanical insult of 100 cycle bending at *r*: 8 mm, there were evident crack propagation in the conventional ITO products and the active layers on top, demonstrating the brittle nature of ITO films; whereas no obvious crack propagation appeared in the flexible PEDOT:PSS anodes in the flexing tests for 2500 cycles. As a result, the CP‐based OSCs fabricated on glass and PET substrates yielded a high PCE of 3.27% and 2.80%, respectively. Meanwhile, the flexible solar cells retained most (≈96%) of the initial efficiency in over 300 cycle bending tests. Therefore, the conductive PEDOT:PSS anodes with low cost had a grand potential for making flexible PVs as a promising alternative of expensive and brittle ITO products.

Bao et al.^67^ made a pioneering research in terms of flexible OSC fabrication. A stretchable and conductive PEDOT:PSS film (Clevios PH1000) was demonstrated through the additive treatments of DMSO and fluorosurfactant Zonyl FS‐300. On the basis of the PEDOT:PSS anodes, the flexible OSCs with P3HT:PCBM active layers *yielded* a considerable PCE of 2.22% compared to that of the ITO electrode–based device.^139^ Kaltenbrunner et al.^165^ demonstrated a new configuration of flexible organic solar cells featuring ultrathin and lightweight. In the devices, conductive PEDOT:PSS films were coated on 1.4‐µm‐thick PET substrates that were prior supported by a glass slide coated with PDMS. Note that the PEDOT:PSS anodes with 100 Ω sq^−1^ were obtained from the solutions (Clevios PH1000, mixed with 5 vol% DMSO and 0.5 vol% Zonyl‐FS300). A flexible OSC (total thickness <2 µm) with the CP electrodes was thus demonstrated, showing the highest PCE of 4.2% among the P3HT:PCBM‐based flexible OSCs.

The electrical conductivity of the PEDOT:PSS films should be considered with respect to the fabrication of efficient, flexible, and ultrathin PVs. The secondary solvent‐modified PEDOT:PSS electrodes mentioned above just showed a moderated conductivity of 500−1000 S cm^−1^. In 2012, Ouyang et al.^78^ made a significant breakthrough in terms of the conductivity of PEDOT:PSS films. The conductivity of PEDOT:PSS films was substantially boosted to 3000 S cm^−1^ via 1.0 m H_2_SO_4_ soaking treatments at ≈160 °C. ITO‐free OSCs fabricated on the PEDOT:PSS/glass substrates yielded a high PCE of 3.5% through using the typical active layer of P3HT:PCBM, which was comparable to that of rigid solar cells with ITO‐coated glass substrates. Based on this, Kim et al.^79^ moved forward with the H_2_SO_4_ soaking modification that enabled a further improvement in conductivity of PEDOT:PSS films. 100% concentrated H_2_SO_4_ was employed to soak as‐cast films, leading to the formation of highly crystallized PEDOT nanofibrils with structural rearrangement. Thanks to the best performing PEDOT:PSS electrodes with a sheet resistance of 46.1 Ω sq^−1^ and 90% transparency at λ = 550 nm, ITO‐free OSCs showed a PCE as high as 6.6%. In views of the pioneering researches, strong acid treatments are considered as the most effective way to dramatically improve the CP conductivity by three orders of magnitude (from 1 to over 3000 S cm^−1^). However, such harsh conditions (i.e., long‐term strong acid soaking and high‐temperature curing) are incompatible with most plastic underlying substrates,^81^, ^131^ owing to the chemical/physical reactions between strong acids and plastic substrates, leading to a large loss in flexibility and transmittance of the underlying plastic substrates.

**Figure 17:**
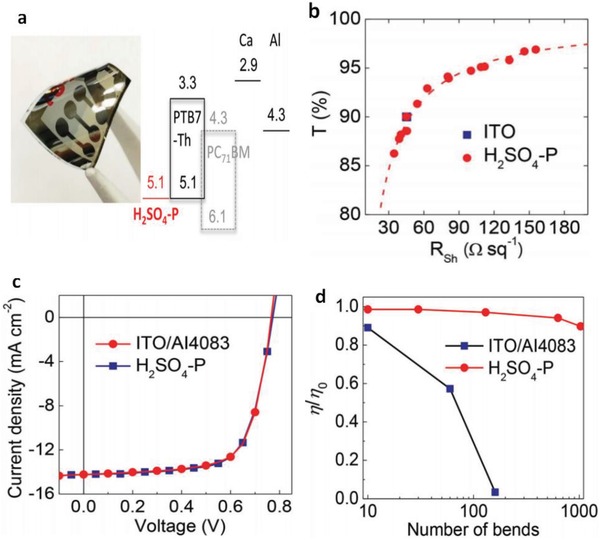
a) Photography and energy‐level diagram of the flexible devices fabricated on PEN substrates. b) Transmittance (at 550 nm) as a function of sheet resistance for the PEDOT:PSS anodes. c) Comparison of the *J–V* characteristics of the flexible OSCs with PEDOT:PSS anodes and ITO anodes. d) Normalized efficiencies for the flexible devices versus the bending cycles at *r* of 1 mm. Reproduced with permission.^80^ Copyright 2015, Wiley‐VCH.

To solve the issue, Lee et al.^80^ demonstrated a unique transfer‐printing technique assisted by 98 wt% H_2_SO_4_. With the H_2_SO_4_ treatment, highly conductive PEDOT:PSS films were feasibly transfer‐printed from rigid substrates to plastic substrates. As shown in **Figure** **17**, the H_2_SO_4_‐treated PEDOT:PSS FTEs on polyethylene naphthalate (PEN) plastic substrates exhibited a low sheet resistance (45 Ω sq^−1^) along with high optical transmittance in the visible region (>90%). The flexible OSCs with the transfer‐printed PEDOT:PSS anodes and PTB7‐Th:PC_71_BM active layers yielded a high PCE up to 7.7%, which is much close to that of conventional ITO‐based OSCs. Note that the flexible OSCs had an outstanding mechanical compliance, retaining beyond 90% of the initial PCE in 1000 cycle bending tests at a bending radius as low as 1.0 mm. Otherwise, the HNO_3_‐assisted transfer‐printing method was also developed to enable efficient semitransparent solar cells. A highly conductive PEDOT:PSS film treated by HNO_3_ was transfer‐printed as the top transparent electrode.^179^ Moreover, Fan et al.^81^ developed a weakly destructive transfer‐printing method assisted by MSA and phosphoric acid (H_3_PO_4_). The gentle method avoids the common drawbacks caused by the usage of 100% concentrated H_2_SO_4_, that are, the large loss in film flexibility and device stability and the concerns of safety and environments. On the basis of the PEDOT:PSS electrodes, the flexible OSC yielded a PCE of 6.42%, and it maintained the most (≈84%) of the initial PCE in 30 day storage in N_2_‐filled glove‐boxes. The enhanced device stability was attributed to less mild acid residues on anode surfaces and the gentle property of these acid residues. Despite the numerous methods used to transfer‐print the CP films, it is challenging to make high yields of large‐area products (>3 cm × 3 cm) without pinholes and cracks, mostly due to the requirement of precisely adjusting the VDW interactions among the rigid donor substrates, PDMS mediums and plastic target substrates. We envision that the efficiency of the CP‐based flexible OSCs with active area of 1.0 cm^2^ will exceed 12.0% over the next two years through the combination of highly conductive and smooth CP electrodes without visible micromorphological defects and more efficient active layers with low band‐gap conjugated polymers.

#### 5.1.2 Flexible OSCs with Hybrid FTEs

**Figure 18:**
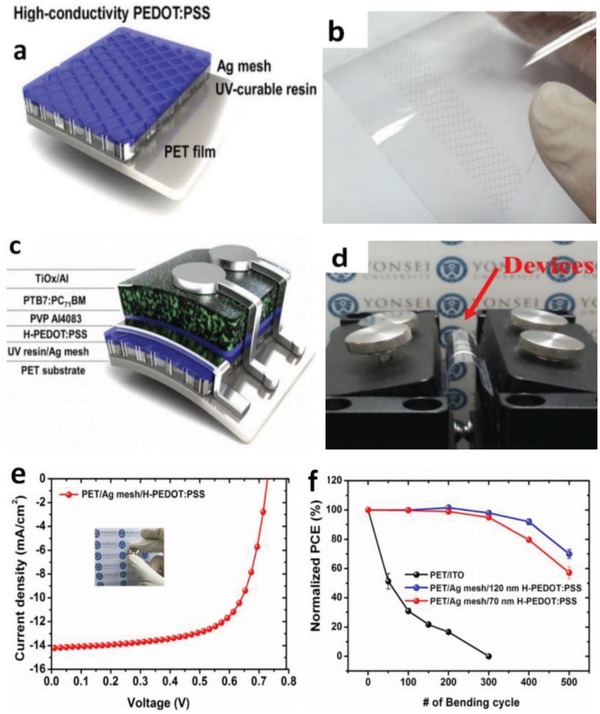
a) Schematic diagram and b) photography of PET/Ag mesh/PEDOT:PSS hybrid electrodes. c) Schematic diagram of the flexible OSCs. d) photography of device bending. e) *J–V* characteristics of the OSCs. f) Normalized PCE decay of the flexible OSCs with ITO, PEDOT:PSS, and Ag mesh/PEDOT:PSS hybrid electrodes, respectively, in a continuous flexing test for 500 cycles. Reproduced with permission.^173^ Copyright 2016, Wiley‐VCH.

Hybrid components open a venue to realize high‐performance FTEs due to the formation of robust composites with enhanced interface adhesion and the multiconductive pathways provided. The metallic NW and grid electrodes tend to have rather large roughness that hinders device fabrication and integration. Park et al.^173^ demonstrated a hybrid anode of Ag mesh/highly conductive PEDOT:PSS that exhibited a *R*_sh_ of 10 Ω sq^−1^ and 74% transparency. The fabricated OSCs with PTB7:PC_71_BM showed a high fill factor (FF) of 67.11%, accounting for the PCE as high as 6.94%. The efficiency is comparable to that of the solar cells with a typical ITO electrode (**Figure** **18**). It is attributed to the smooth surfaces of hybrid electrodes, good adhesion between Ag meshes and PEDOT:PSS as well as the current homogeneity, leading to an effective charge‐carrier collection by the discrete Ag meshes. Furthermore, the flexible devices showed excellent mechanical bending stability, retaining 70% of the initial PCE in flexing tests for 500 cycles. However, the electrode roughness is as high as 19.3 nm, perhaps causing an electric short‐circuiting despite the coating of PEDOT:PSS films used to smooth the Ag mesh surfaces.

**Figure 19:**
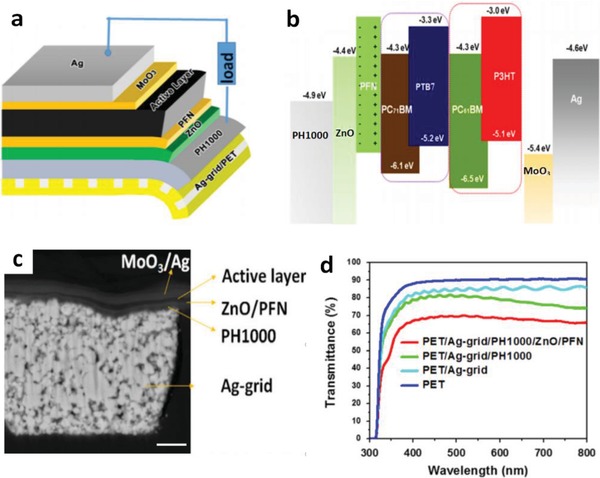
a) Device structure of the flexible inverted OSCs. b) Energy levels of the materials in the device. c) Cross‐sectional SEM images of inverted OSCs. Scale bar: 1.0 µm. d) Transmittance spectra of the samples including PET, PET/Ag‐grid, PET/Ag‐grid/PH1000, and PET/Ag‐grid/PH1000/ZnO/PFN films. Reproduced with permission.^180^ Copyright 2014, Elsevier.

**Figure 20:**
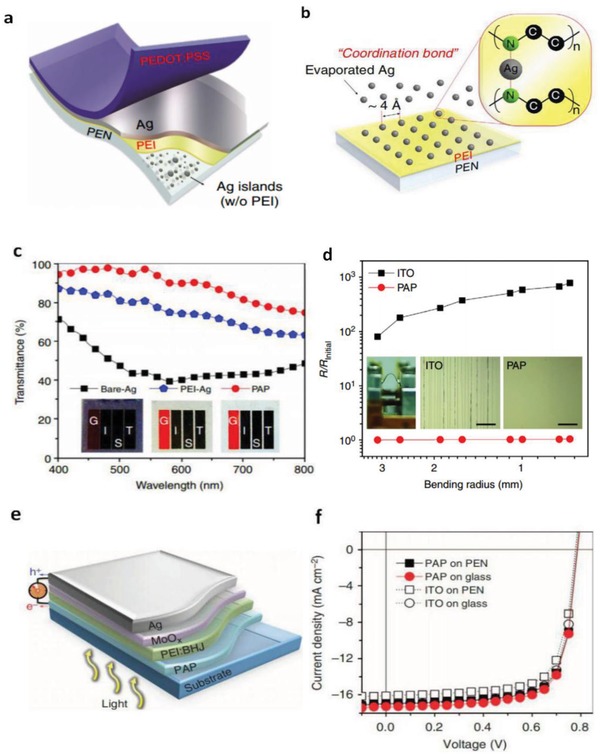
a) Flexible PAP electrodes consisting of the ultra‐thin Ag film sandwiched between PEI and PEDOT:PSS. b) Diagram of the growth mechanism of the Ag film with the PEI nucleation inducer. c) Optical transmittance of three kinds of electrodes including Ag film, PEI‐Ag, and PAP. Insert: an electrode located on GIST logos. d) Increase in resistance versus the bending radius of the PAP and the commercial ITO. e) Device architecture. f) *J−V* characteristics of the OSCs. Reproduced with permission.^19^ Copyright 2018, Nature Publishing Group.

Chen et al.^180^ reported an innovative hybrid transparent electrode incorporating high‐resolution Ag grids and PEDOT:PSS (Clevios PH1000) (**Figure** **19**). The hybrid electrodes showed a considerable transparency of 78% at λ = 550 nm and a much low sheet resistance of ≈1 Ω sq^−1^. On the basis of PTB7:PC_71_BM active layers, the normal flexible OSCs with 1.0 cm^2^ active area showed a high efficiency of 5.85%, which was stark contrast to the flexible devices with conventional ITO that exhibited a limited efficiency of 4% yet. The enhanced efficiency is attributed to both factors below: 1) extremely low sheet resistance of the PEDOT:PSS/Ag‐grid hybrid electrodes for efficient charge‐carrier collection, and 2) the smooth surfaces of the hybrid electrodes for device integration. To our best knowledge, the hybrid transparent electrodes demonstrated the best‐performing merit in *R*_sh_ among the FTEs reported, so far. The hybrid films are very suitable to fabricate other types of solar cells and OLEDs with high efficiency and large size required by next‐generation printed PV modules. For the adaptation of PEDOT:PSS to OSC modules, Kang et al.^19^ reported a hybrid electrode of polyethyleneimine (PEI)/ultrathin Ag (<10 nm)/PEDOT:PSS (namely PAP), as shown in **Figure** **20**. Through the combination of the PEI nucleation inducer and the CP anti‐reflective layer, the state of the art transparent hybrid electrode was prepared. It possessed *R*_sh_ < 10 Ω sq^−1^, high transmittance of ≈95% at λ = 550 nm and extreme flexibility. Notably, the transmittance of PAP hybrid electrode is higher by 20% as compared to the the PEI‐Ag electrode. The OSCs exhibited an outstanding PCE of 10%. Krebs et al.^181^ demonstrated printed organic solar cell modules that exhibited a considerably high efficiency of 2.3% with a total active area of 8 cm^2^. In the case of the operational stability of devices, the solar cell modules based on Ag NW/PEDOT:PSS electrodes generally retained their initial efficiency under constant illumination at 60 °C. However, an increased temperature of 100 °C led to a rapid decrease in performance of the devices, that was, the device efficiency dropped to 20% within 250 h. Thus, the heat stability of the modules still needs to be enhanced further.

The aforementioned modifications and strategies for making high‐merit FTEs can extend to encompass a variety of flexible and semitransparent optoelectronics (e.g., OLEDs, thermoelectrics and touch sensors). Some excellent research articles on OLEDs,^27^, ^28^, ^29^, ^30^, ^31^, ^32^, ^33^ thermoelectrics,^45^, ^46^, ^47^, ^48^, ^49^ and touch sensors^56^, ^93^ are recommended to interested readers. **Table** **4** shows the photovoltaic characteristics of ITO‐free flexible OSCs based on PEDOT:PSS electrodes and their hybrid electrodes.

**Table 4:** Photovoltaic characteristics of ITO‐free flexible OSCs using PEDOT:PSS electrodes and the hybrid electrodes

Device structure	*V*_OC_	*J*_SC_	FF	PCE	Retained (PCE)	Flexing test	Reference
PET/PET/Ag‐grid/PEDOT:PSS(PH1000)/PTB7:PC_71_BM/LiF/Al	0.7	13.7	61	5.85	–	–	180
PET/Ag‐grid/PEDOT:PSS(PH1000)/ZnO/PFN/active layer/MoO_3_/Ag	0.72	13.9	60	6.01	–	–	180
PEN/H_2_SO_4_‐PEDOT:PSS(PH1000)/PTB7‐Th:PC_71_BM/Ca/Al	0.77	14.2	69	7.6	90%	10^3^ cycling, *r*: 1 mm	80
PEN/PEI/Ag/PEDOT:PSS(PH1000)/PTB7‐Th:PC_71_BM/MoO_3_/Ag	0.79	16.9	74	9.9	–	–	19
PET/Ag‐mesh/PEDOT:PSS/AI4083/PTB7:PC_71_BM/TiO*_x_*/Al	0.73	14.2	67.1	6.94	65%	10^3^ cycling, *r*: 5 mm	173
PET/Ag‐grid/PH1000/ZnO/PTB7‐Th:PC_71_BM/MoO_3_/Al	0.78	14.3	59	6.58	–	–	174
PES/PH1000/PEI/P3HT:ICBA/AI4083/PEI/P3HT:ICBA/PEDOT‐T	1.55	7.0	59	6.1	88–90%	5 × 10^3^ cycling, *r*: 4 mm	20
PET/Acid‐PH1000/AI4083/P3HT:PCBM/Ca/Al	0.595	10.2	65	3.92	80%	100 cycling, *r*: 14 mm	125
PET/Acid‐PH1000/AI4083/PBDB‐T:IT‐M/Ca/Al	0.93	15.5	70.3	10.12	94%	10^3^ cycling, *r*: 5.6 mm	131

#### 5.1.3 Flexible OSCs with PEDOT:PSS Composite Buffer Layers

**Figure 21:**
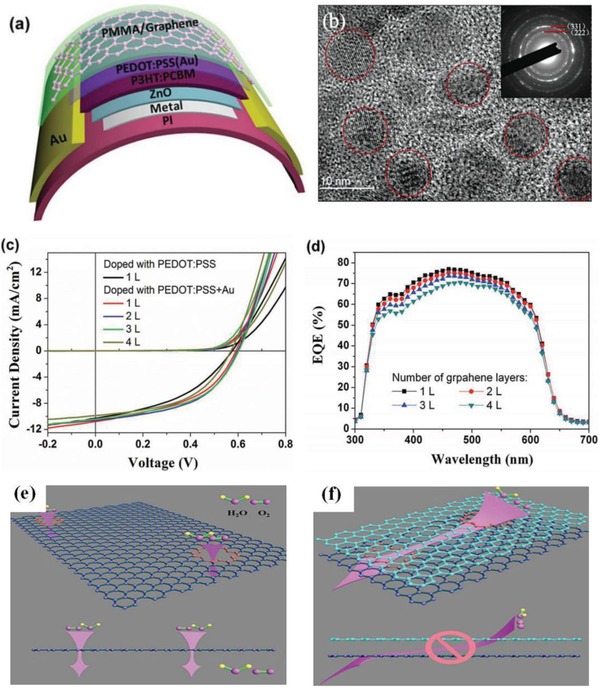
a) Schematic diagram of the flexible OSCs. b) TEM image of Au NPs in PEDOT:PSS matrices. c) *J−V* characteristics of the OSCs with different number of layers of graphene anode doped with PEDOT:PSS+Au or PEDOT:PSS only. d) EQE of the devices with different number of layers of graphene anodes. Schematic diagram of e) single‐ and f) double‐layer graphene layers for blocking H_2_O and O_2_ penetration. Reproduced with permission.^162^ Copyright 2013, Wiley‐VCH.

Besides the role as a transparent electrode, the PEDOT:PSS films can serve as a hole transport layer (HTL) for PVs and an hole injection layer for light‐emitting diodes. By increasing the conductivity of the HTLs, it is favorable to achieve a higher efficiency for flexible OSCs. For instance, Yan et al.^162^ demonstrated a package‐free flexible OSC with a HTL consisted of PEDOT:PSS and Au nanoparticles (NPs) (**Figure** **21**). The flexible OSC had a device structure of PI/Metal/ZnO/P3HT:PCBM/PEDOT:PSS (Au)/graphene/PMMA. The single‐layer graphene film modified by the conductive HTL showed a decreased sheet resistance of 158 Ω sq^−1^. The Au NPs in PEDOT:PSS films were also used to enhance the doping effect in graphene due to a decrease in Fermi energy for the graphene films.^163^ The flexible OSCs with P3HT:PCBM yielded the maximum PCE of 3.2% and excellent bending stability, and the devices had a slight degradation in PCE by 8% in 1000 cycle bending tests at *r* of 1.5 mm. Meaningfully, two or more layers of graphene top electrodes also protected the PEDOT:PSS layers from the contamination of air because multilayer graphene films were impermeable to air.

### 5.2 ITO‐Free and Flexible PSCs

#### 5.2.1 Flexible PSCs with PEDOT:PSS Electrodes

PSCs have received worldwide attention since Miyasaka et al.^182^ first reported a prototype working device with a PCE of 4% in 2009. The PCE of PSC devices has exceeded 23% to date,^183^, ^184^ which was mostly attributed to a long exciton diffusion length (>1000 nm) of perovskites, high absorption coefficient and a favorable band gap (1.55 eV). Up to now, the PCE of flexible ITO‐based and ITO‐free PSCs with rapid developments had been boosted to 18.4%^185^ and 16.8%,^178^ respectively. However, ITO‐based PSCs suffered from a limited mechanical flexibility due to the brittle and fragile natures of bulk ITO films. The modified CP electrodes had been used to fabricate ITO‐free flexible perovskite solar cells as an alternative of ITO. For example, Qiao et al.^186^ presented a perovskite solar cell using highly conductive PEDOT:PSS films. The CP films were modified by the secondary dopants (i.e., DMSO and EG) followed by oxygen plasma treatments. The synergistic effect of DMSO and EG facilitates to the rearrangement of PEDOT:PSS, and it is definitely favored to a removal of excess insulating PSS chains from PEDOT:PSS matrices. The PEDOT:PSS electrodes thus showed a sheet resistance of 85 Ω sq^−1^ with an average transmittance of 73% in the range of 350−900 nm. With the subsequent oxygen plasma treatment, the CP electrodes exhibited a sheet resistance as low as 36 Ω sq^−1^ with a raised wettability. As a result, the as‐fabricated PSCs with an architecture of glass/PH1000/AI4083/pervoskite/PCBM/rhodamine/Ag yielded a high efficiency of 10.5%. The high value is comparable to that of the devices with similar architecture reported by Sun et al.^187^ In the literature,^187^ a PEDOT:PSS film was treated by MSA at high temperatures of ≥130 °C, and then it is employed as the anode of PSCs. The results demonstrated that double layers of PEDOT:PSS had a considerable conductivity of 2540 S cm^−1^ and a high transmittance of 92% at λ = 550 nm. The WF of PEDOT:PSS electrodes was increased from −5.0 to −4.8 eV due to the acid modification, which was energetically favorable for charge‐carrier transport from the buffer layers to PEDOT:PSS anodes and collection by the anodes. Therefore, ITO‐free PSC devices with glass and PET substrates yielded a high average efficiency of 10.6% and 8.01%, respectively. It should be notable that the flexible PSCs with PET substrates showed a degradation in efficiency from 7.5% to 5.5% in bending tests at 500 cycles (bending radius: 2−3 mm). The work demonstrated the visible enhancement in efficiency of the flexible PSCs through employing acid‐treated PEDOT:PSS transparent anodes, and it provided a significant route to make highly efficient and mechanically flexible PV devices with PEDOT:PSS anode materials.

The PEDOT:PSS anodes are also suitable to fabricate flexible PSCs featuring semitransparent characteristics. For example, Zhang et al.^179^ demonstrated a proof‐of‐concept of flexible semitransparent ITO‐free PSCs with transfer‐printed PEDOT:PSS films as top and bottom electrodes. The resulting solar cells yielded a high efficiency of 10.3% along with high bending flexibility. Moreover, over 90% of the initial PCE was maintained in the flexible devices after 1000 cycle bending at *r* of 5 mm. The optical transmittance of the whole devices is ≈60% at λ = 800 nm. However, the surfaces of PET substrates used seem to be rough. It is advised that polymeric products such as ZEOCOAT ES2110^188^ and cross‐linked polymers are used to smooth the PET surfaces and raise the substrate wettability for an intimate interface contact.

It is also critical to develop photovoltaic technologies toward ultrathin, lightweight, and stable PV devices. Kaltenbrunner et al.^31^ reported ultrathin (only 3 µm), highly flexible PSCs that showed a stabilized 12% efficiency and a high power‐per‐weight of 23 W g^−1^. The PEDOT:PSS anodes were prepared from stock Clevios PH 1000 with 5 vol% DMSO and 0.5 vol% Zonyl FS‐300 fluorosurfactant. Due to 5% DMSO doping, closed and uniform CP films with less pinholes were obtained. In the case of device fabrications, a glass slide coated with PDMS (so‐called PDMS/glass) served as a supporting layer. The 1.4‐µm‐thick PET foil was adhered onto PDMS/glass via VDW interactions, enabling the next processing steps (i.e., spin coating, thermal annealing and vacuum evaporation). For a better device stability, a chromium oxide–chromium interlayer (Cr_2_O_3_/Cr) was introduced to shield the top contact metal from detrimental reactions with oxidizing and halide‐forming iodide species. Due to the weak VDW interactions, the solar cell foils were prone to peeling off from the supporting layers of PDMS/glass. As a result, the PV foils covered by a spray‐painted PU resin operated stably in ambient air, showing a stable PCE of 12% over 1000 s and dropped with a plateau of 8.0% efficiency over 5 × 10^4^ s.

#### 5.2.2 Flexible PSCs with Hybrid FTEs

**Figure 22:**
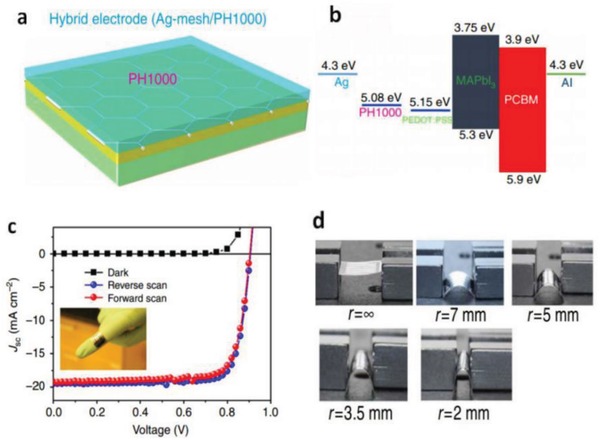
a) Schematic diagram for the hybrid electrode (PET/Ag‐mesh/PH1000). b) Energy‐level diagram of the materials used. c) *J–V* curves in reverse and forward. d) Bending and stability test of flexible PSCs at different bending radii. Reproduced with permission.^189^ Copyright 2016, Nature Publishing Group.

Hybrid transparent electrodes commonly have the advantages of low *R*_sh_, highly mechanical flexibility, and smooth surfaces for the intimate contacts with other layers grown on top. Hybrid transparent electrodes tend to be predominant products for making high‐efficiency flexible PV devices with high mechanical compliance. Li et al.^189^ reported flexible PSC devices with highly flexible and ultrathin Ag mesh/PEDOT:PSS (PH1000) that yielded a PCE of 14.0%, which is the record‐high PCE among these ITO‐free flexible PSCs based on PEDOT:PSS anodes (**Figure** **22**). The flexible device exhibited a high mechanical flexibility, retaining ≈95% of its initial PCE after 5000 cycle bending. However, non‐uniform thermal conductivity of the hybrid electrodes perhaps led to spatially non‐homogeneous conversion of the perovskite precursor, thereby hampering the formation of high‐quality perovskite films on top.^178^

More recently, Ha and Noh et al. demonstrated a hybrid FTE that consisted of metallic grids and PEDOT:PSS for making durable PSCs. Cr (≈3 nm) and Au (≈60 nm) were deposited on the surfaces of NOA63/PI substrates. Note that NOA63 has the advantages of affording high‐temperature thermal annealing and mechanical deformations over the conventional plastic substrates such as PET and PEN.^190^ Using photolithography and etching methods, Cr/Au grids were patterned. Then, PEDOT:PSS (Clevios PH1000) doped with 5 wt% EG was spin‐coated on the metallic grid electrodes for the obtainment of hybrid FTE films of Cr/Au grid/PEDOT:PSS. The hybrid electrodes with the NOA63/PI substrate exhibited a high transparency of 90.7% at λ = 550 nm and *R*_sh_ of 30.3 Ω sq^−1^, and the films withstood both moderate thermal annealing at 180 °C and harsh acid processing (70 °C, pH: 0.3) without obvious performance degradation. The flexible PSCs showed a steady‐state PCE of 12.7%, retaining 93% of the original PCE after 2000 bending cycles at an extremely small bending radius of 1.5 mm. There is an urgent need for the desirable hybrid electrode that is electrically conductive, highly transparent, spatially homogeneous, and durable against each mechanical insult. **Table** **5** compares the photovoltaic characteristics of the PSCs based on PEDOT:PSS, hybrid electrodes, graphene, ITO, respectively.

**Table 5:** Photovoltaic characteristics of the flexible PSCs with PEDOT:PSS, hybrid electrodes, graphene, and ITO, respectively

Device structure	*V*_OC_	*J*_SC_	FF	PCE [%]	Retained PCE [%]	Flexing test	Reference
PET/PH1000/ZnO/perovskite/Spiro‐OMeTAD/PH1000	0.99	17.3	60	10.3	>90	10^3^ cycling, *r*: 5 mm	179
PET/PH1000/perovskite/PCBM/PTCDI/Cr_2_O_3_/Cr/Au/polyurethane	0.93	17.5	76	12	100	50% compression	31
PET/PH1000/AI4083/perovskite/PCBM/rhodamine/C_60_/rhodamine/LiF/Ag	0.86	17.2	57	8.6	73	500 cycling, *r*: 2–3 mm	187
PET/Ag‐mesh/PH1000/AI4083/perovskite/PCBM/Al	0.91	19.5	80	14	96	10^3^ cycling, *r*: 5 mm	189
PET/PH1000/MAPbI_3_/TiO_2_/Al	0.80	15.0	60.0	7.60	47	50 cycling, *r*: 4 mm	22
PET/PH1000/PEI/MAPbI_3_/Spiro‐OMeTAD/Au	0.95	17.2	59.7	9.73	75–80	500 cycles, *r*: 3 mm	23
NOA63/PH1000/MAPbI_3_/PCBM/eutectic Ga‐In blends	0.92	16.6	70.5	10.75	80	50 cycles, *r*: 6 mm	24
PEN/graphene‐Mo/AI4083/MAPbI_3_/C_60_/BCP/LiF/Al	1.0	21.7	80	16.80	>90	10^3^ cycling, *r*: 2 mm	178
PET/ITO/TiO_2_/MAPbI_3−_ *_x_*Cl*_x_*/Spiro‐OMeTAD/Au	1.03	20.9	70.0	15.07	>95	100 cycling, bending angle: 65°	26
PEN/ITO/AI4083/MAPbI_3_/C_60_/BCP/LiF/Al	0.97	21.5	83.0	17.30	54	10^3^ cycling, *r*: 4 mm	178
PEN/ITO/SnO_2_/CsMAFA/Spiro‐OMeTAD/Au	1.11	20.9	73	16.97	–	–	25
PEN/ITO/Nb_2_O_5_/MAPbI_3_–DS/Spiro‐OMeTAD/Au	1.10	20.8	80	18.4	83	5 × 10^3^ cycling, *r*: 14 mm	185

#### 5.2.3 Flexible PSCs with PEDOT:PSS‐Based HTLs

PEDOT:PSS (AI4083) combined with metal oxides are suitable to flexible PV devices as a HTL. Choi et al.^178^ demonstrated a highly efficient and flexible PSC with molybdenum oxide (MoO_3_) and PEDOT:PSS (AI4083) component HTLs. Here, 2 nm‐thick MoO_3_ films were sandwiched between graphene electrodes and the PEDOT:PSS films (AI4083). The thin layers of MoO_3_ not only induce hole doping in the graphene,^31^ but also enable the graphene to be hydrophilic. The wettability of the PEDOT:PSS droplets on graphene surfaces was thus substantially improved. The device exhibited an efficiency of 16.8% without hysteresis, which was comparable to that (17.3%) of the flexible devices with ITO electrodes. The flexible solar cells also showed high stability against bending deformations, retaining 90% of the original PCE for 1000 bending cycles and 85% for 5000 bending cycles at *r* of 2 mm. Chu et al.^191^ fabricated the flexible PSC devices through the deposition of ultrathin MoO_3_ layers on PEDOT:PSS HTLs. Introducing the ultrathin MoO_3_ layer resulted in the alignment of energy levels and efficient charge‐carrier extraction. By varying the thickness of the MoO_3_ layer from 0 to 6 nm, the WF of PEDOT:PSS was decreased to −5.10 eV. The resulting PSCs achieved a high PCE of 13.54% without hysteresis. Brabec et al.^192^ reckoned that the PEDOT:PSS/MoO_3_ components were able to overcome interface losses in organic multijunction solar cells through reducing the interface protonation, aligning the energy levels and forming a dense and smooth HTL.

Typically, there was a phenomenon of exciton quenching occurred at the interface between PEDOT:PSS (AI4083) HTLs and metal halide perovskite, thus delaying the device efficiency by a radiative recombination of charge carriers.^193^, ^194^, ^195^ Via increasing the work function of PEDOT:PSS layers, it provides a pathway for making efficient perovskite solar cells. For instance, Ding et al.^196^ reported that the regular PEDOT:PSS (AI4083) was treated by adding the polymer electrolyte (i.e., PSS‐Na) to improve its work function. Compared to that of the regular one, the work function of the treated PEDOT:PSS film (namely m‐PEDOT:PSS) was higher by 0.3 eV. Its energy level matched well the valence band of perovskite layers, resulting in an enhanced open circuit voltage (*V*_OC_) and PCE. It had been found that the HTLs added with PSS‐Na are energetically favored to fabricate high‐efficiency PSC devices with a high *V*_OC_. Otherwise, a commercial product of PEDOT:PSS (Clevios P CH8000) had a lower conductivity and higher work function than AI4083. The PSS enriched top surface forms an insulating layer, not only preventing exciton quenching, but also blocking the hole currents at the interface between PEDOT:PSS HTLs and active layers.^197^, ^198^ Moreover, the typical WF of PEDOT:PSS (AI4083) is −4.85 eV; whereas the WF of PEDOT:PSS (Clevios P CH8000) is as high as −5.15 eV,^199^ favoring the hole transportation in PV devices.

In the case of organic/perovskite PV fabrications, it takes intensive researches to compete against existing silicon solar cells that have dominated the PV markets till now. A wide range of studies including conductive material modification, interface shields, and device fabrication/integration should be organically performed to enhance device efficiency, flexibility, and lifetime for inventing their future.

## 6 Stretchable Electronics

Stretchable devices such as stretchable OSCs/PSCs, OLEDs, OTFTs, and bio‐, strain‐, and touch sensors all are regarded as the next‐generation ubiquitous electronics. These devices rely on a stretchable conductor and have to operate stably under considerable tensile strains. The most successful concept leading to such devices builds on the fabrication of transparent electrodes for stretchable PVs, motion‐sensing conductors for strain sensors, linking rigid islands of each device for transistors, etc.^134^, ^200^, ^201^ Indeed, PEDOT:PSS is the most commonly used stretchable conductor material and plays a key role in these devices. A variety of strategies have been developed to make the unprecedented stretchable electronics with PEDOT:PSS.

### 6.1 Strain Sensors

#### 6.1.1 Strain Sensors with PEDOT:PSS Conductors

Strain sensors commonly consist of a stretchable conductor and an elastomer as underlying substrates or blends. The stretchable conductors are deformed under external mechanical forces and have a resistance‐tunneling channel, thus generating an output of electrical signals. The strain sensors thus are capable of distinguishing diverse deformations of target objects and have promising applications as electronic skins, health monitors, soft robotics, etc.^202^, ^203^, ^204^, ^205^, ^206^ Differing from other strain sensors based on metallic NWs, metallic films, and CNTs, the plastic strain sensors based on PEDOT:PSS conductors have the combined advantages such as comfortable attachment above epidermis, various geographical shapes/configurations, cost‐effective fabrication, and biocompatibility. The plastic sensing devices are scalable to make epidermal electronics and implantable sensors.

**Figure 23:**
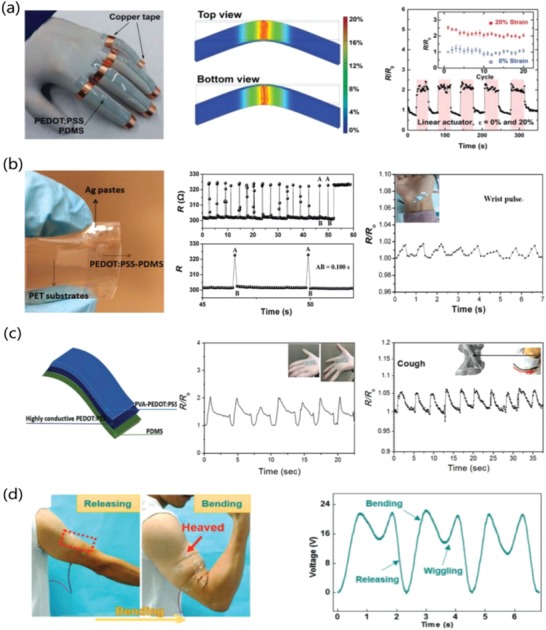
Strain sensors with PEDOT:PSS. a) Device photograph and computational analysis of the device subjected to bending with *r* of 5 mm. *R*/*R*_0_ versus time for the devices with a linear actuator cycling at 20% strain. Reproduced with permission.^151^ Copyright 2016, Wiley‐VCH. b) Response of strain sensors to cyclic bending by the fingers; replot of real‐time wrist pulse waves. Reproduced with permission.^60^ Copyright 2016, Wiley‐VCH. c) Schematic diagram of the sandwiched devices. Monitoring of the motions of palm and detection of the cough. Reproduced with permission.^210^ Copyright 2016, Royal Society of Chemistry. d) Schematic of the motion sensor fixed at the elbow to monitor the bending amplitude. Reproduced with permission.^211^ Copyright 2016, Wiley‐VCH.

However, the plastic strain sensors with PEDOT:PSS stretchable conductors suffered from poor sensitivity and response reliability issues along with small strain‐sensing regions due to the slippages of conducting components and the viscoelasticity of underlying elastomers.^147^, ^148^, ^207^, ^208^ Lopomi et al.^151^ reported an elastomeric strain sensor that consisted of PEDOT:PSS and PDMS (**Figure** **23**a). The modified CP films were prepared from the PEDOT:PSS aqueous solution (Clevios PH1000) incorporating 5% DMSO and 10% Zonyl FS‐300. Wearable electronic sensors with the PEDOT:PSS films exhibited a sensitivity of ≈5.5 for 20% strain. Note that the sensitivity (also namely gauge factor [GF]) was obtained by GF = (Δ*R*/*R*_0_)/ε. Here, Δ*R*/*R*_0_ is the normalized change in electrical resistance, and ε is the tensile strain of strain sensors. In a previous literature,^209^ the strain sensors consisting of a PEDOT:PSS film modified by DMSO and a PET substrate also showed a limited sensitivity of ≈2.5% at 20% strain. For making a sensitive and large‐area strain sensor, Fan et al.^60^ developed a dipping‐embedded transfer‐printing technology that enabled a highly conductive and stretchable PEDOT:PSS embedded into PDMS elastomers (namely PEDOT:PSS–PDMS) (Figure 23b). The PEDOT:PSS–PDMS films had less insulating PSS chains and more refined PEDOT nanofibrils due to the usage of MSA modification, thereby resulting in a large change in resistance under strains and a high sensitivity of 22 for 20% strain without obvious signal fluctuation/noise under release states. A large‐area (4.7 cm × 4.7 cm) stretchable PEDOT:PSS film was presented as well. When the smart sensor was attached onto human wrists, the resistance responses for pulse wave detection were obtained effectively before and after exercise. The wrist pulses were read out accurately under both conditions (i.e., 68 and 86 beats min^−1^ before and after exercise). However, the small strain‐sensing region (<20%) and stability issue hamper the developments of the soft strain sensors. For moving forward with PEDOT:PSS adoption into wearable motion‐sensing electronics, Fan et al.^210^ demonstrated a soft strain sensor with a sandwiched structure of PVA–PEDOT:PSS blends/highly conductive PEDOT:PSS/PDMS (Figure 23c). The wearable strain sensors exhibited an enhanced sensitivity of 26 for 10% strain and 110 for 30% strain, and the devices obtained a stable response in both repeated stretching–relaxing and long‐time (over 120 s) loading tests at 30% strain. The high sensitivity and response stability were attributed to the formation of fine crack propagation of the sandwiched films and the physical connections between cracks soldered by the PVA modified PEDOT:PSS films when the devices were relaxed.

A wrinkled design provides a large strain‐sensing ability for strain sensors. Wen et al.^211^ demonstrated a wearable strain senor through using a highly stretchable and transparent wrinkled PEDOT:PSS film. The process of device fabrication along with response behaviors are shown in Figure 23d. With the large strain of 100%, the PEDOT:PSS films by blade coating showed a wave‐like structure with an inner wrinkle distance of 5 µm and a thickness of 0.74 µm. The device was employed to monitor human motions such as detecting the bending angles of the elbow and joint, and mapping the touch location or recording the shape of object contacted with the sensor array. Its potential for PEDOT:PSS applications into a variety of fields including artificial e‐skins and health monitors.

#### 6.1.2 Strain Sensors with Hybrid Motion‐Sensing Materials

**Figure 24:**
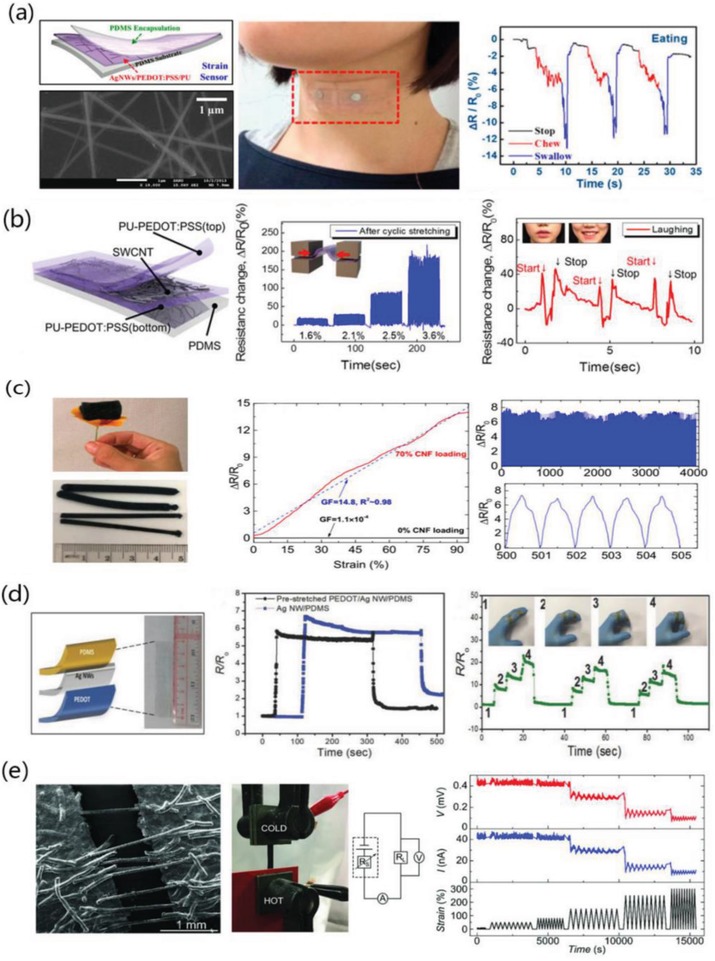
Hybrid strain sensors. a) Ag NW/PEDOT:PSS/PU with responses when attached to the neck, sensing swallowing and chowing behavior during eating process. Reproduced with permission.^115^ Copyright 2015, American Chemical Society. b) CNT/PEDOT:PSS/PU with responses to cyclic stretching of different strains and monitoring of laughing behavior. Reproduced with permission.^116^ Copyright 2015, American Chemical Society. c) PEDOT/PSS/CNF aerogels and the durability in stretching–relaxing tests at 50% strain. Reproduced with permission.^213^ Copyright 2013, American Chemical Society. d) PEDOT:PSS/Ag NW/PDMS with reliability measurements and figure motions detection. Reproduced with permission.^149^ Copyright 2018, Wiley‐VCH. e) Self‐powered strain sensors based on PEDOT:PSS:Lycra blends. Reproduced with permission.^49^ Copyright 2018, Wiley‐VCH.

Hybrid motion‐sensing components open a venue to make strain sensors with high performances such as high sensitivity, broad strain‐sensing range, and stable response owing to the combined advantages of each sensing material, the formation of mechanically robust composites, and the strong interface adhesion.^207^, ^212^ For instance, Hwang et al. ever reported a hybrid strain sensor using the multifunctional Ag NW/PEDOT:PSS/PU composites via blending PU solutions with PEDOT followed by the coating Ag NWs (**Figure** **24**a).^115^ The devices exhibited a highly reliable response under small strains (≤6%), which was in stark contrast to Ag NW–based devices. In light of the design of device structure, through blending PEDOT:PSS into PU elastomer followed by spin‐coating the single‐wall CNTs, the CNT/PEDOT:PSS hybrid strain sensors were fabricated (Figure 24b).^116^ The resulting devices showed an enhanced sensitivity up to 49.7 at ε of 3.6% and stable responses thanks to the formation of percolating networks between CNTs and PEDOT:PSS. Thus, the strain sensor was able to detect tiny strains generated by emotional expressions such as laughing, crying, and eye movement of human beings. However, the sensing regions (i.e., ε: 1.6−3.6%) were limited and should be improved further for augmenting their applications. Zhou et al. reported a unique approach to synthesize conductive and robust nanocellulose aerogels by protonating 2,6,6‐tetramethylpiperidine‐1‐oxyl (TEMPO)−cellulose nanofibrils (CNFs) with conductive PEDOT:PSS followed by freezing and EG‐modified these aerogels to produce stretchable strain sensors (Figure 24c).^213^ The EG vapor annealing increased the conductivity of PEDOT/PSS/CNF aerogels by two orders of magnitude. On the basis of the stretchable PEDOT/PSS/CNF aerogels (70 wt% CNF), the strain sensor showed a sensitivity of 14.8 at ε of 95% and a linear response. Fan and co‐workers demonstrated a hybrid strain sensor with a sandwiched structure of PEDOT:PSS/Ag NW/PDMS via a transfer‐printing technique (Figure 24d).^149^ The sandwiched films with large sizes (1.5 × 5.9 cm^2^) were mechanically robust under 30% strain and highly conductive. Owing to a mechanical compliance of the sandwiched films and a compensation of conductivity offered by Ag NWs, the strain sensor with prestraining of 30% showed high response reliability at strains as high as 50% but a limited sensitivity of 6.5−8.0. The devices integrated on fingers distinguished clearly the finger motions at diverse bending deformations.

With respect to the broad applications of PEDOT:PSS, this classic CP material is also regarded as a promising active component of thermoelectric (TE) devices. It is thus feasible to develop the proof‐of‐concept of self‐powered, all‐carbon and cost‐effective strain senors with PEDOT:PSS as motion‐sensing and TE materials. For example, Taroni et al.^49^ demonstrated the viability of the PEDOT:PSS:elastomeric PU (Lycra) blends as stretchable self‐powered strain sensors. The PEDOT:PSS (PH1000 modified by DMSO or EG) was blended with the PU for obtaining a tough and processable self‐standing film. Large strains of beyond 80% were detected visibly through the simultaneous changes in voltage and current (Figure 24e).

**Table** **6** summarizes the sensitivity, sensing region and transmittance of the current strain sensors based on PEDOT:PSS and its hybrids in durability tests. The simple device structures and solution processing of CP‐based strain sensors make the PEDOT:PSS promising for cost‐effective and disposable applications. However, the response stability under cyclic tensile strains remains an obstacle for all that strain sensors. For their practical implementations of the wearable sensing electronics, it is highly desirable and imperative to realize these kinds of strain sensors featuring high stability (i.e., stable response in >10^4^ cyclic stretching–relaxing >30% strain), broad strain‐sensing regions (i.e., ε: 0−100%), optical transparency, ultrathin and water‐proof characteristics.

**Table 6:** Brief summary of current strain sensors based on PEDOT:PSS and the hybrids comprising PEDOT:PSS

Film	Method	GF	Sensing region [%]	Durability test [cycling]	*T* [%]	Reference
PH1000/PDMS	DMSO doping	5	20	>20	–	151
PH1000/PDMS	Transfer‐printing	22	20	400		60
PVA:PH1000/Acid‐PH1000/PDMS	PVA blending and acid treatments	110	30	400	75	210
PU‐PEDOT:PSS/SWCNT/PU‐PEDOT:PSS	PU blending and CNT coating	8.7–62.3	0–3.6	10^3^	62	115
Ag NW/PEDOT:PSS/PU	PU blending and Ag NW coating	5.2–8.3	0–6	10^3^	75	116
PEDOT:PSS/Ag NW/PDMS	Transfer‐printing	6.5–8.0	10–50	300	75	149
PEDOT:PSS/CNF aerogels	EG vapor annealing	14.8 for ε: 0.95	4–100	4 × 10^3^	0	213
PEDOT:PSS (EL‐P3040, Orgacon/PDMS)	Bladed on prestrained PDMS	2 for ε: 1.0	60–100	–	90	211

### 6.2 Stretchable PVs

#### 6.2.1 Stretchable PVs Fabricated on Prestrained Elastomeric Substrates

**Figure 25:**
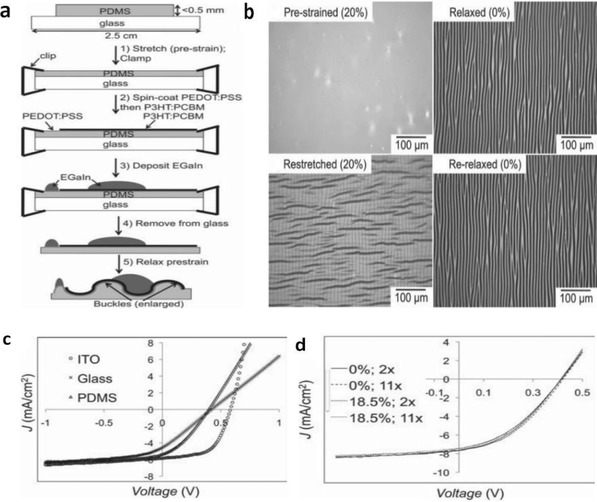
a) Fabrication procedures of stretchable OSCs. b) Optical images of PEDOT:PSS/P3HT:PCBM films on a prestrained PDMS substrate. c) Photovoltaic properties of rigid devices with ITO and PEDOT:PSS electrodes and the stretchable devices. d) Photovoltaic properties of stretchable devices with 11 full cycle 18.5% strain. Reproduced with permission.^214^ Copyright 2011, Wiley‐VCH.

It is not feasible to use ITO in stretchable PVs because ITO is brittle and not compatible with plastic substrates. By coating a modified PEDOT:PSS transparent electrode on prestrained elastomeric substrates, it can enable a stretchable PV with a wrinkled structure. Through the treatment of Zonyl, it improves the wetting behavior of PEDOT:PSS aqueous solutions on plastic substrates and prolongs the elongation of the CP films, leading to the formation of uniform, reproducible, and highly flexible films on hydrophobic substrates. Bao et al. proposed the concept of stretching organic solar cells for the first time (to our best knowledge). These stretchable devices were fabricated through using the PEDOT:PSS anodes that were coated on a strained PDMS substrate.^214^ The droplets of eutectic gallium−indium (EGaIn) were used as the top electrodes,^215^, ^216^ and the Cu wires were fastened into the EGaIn electrodes. Notably, EGaIn is a liquid metal at room temperature so that it can maintain its high electrical conductance when the devices were stretched. The wrinkled PEDOT:PSS films showed a *R*_sh_ of 750 Ω sq^−1^ with 96% transmittance, and did not deteriorate under the strains of no more than 20% for 1000 cycles. **Figure** **25** shows the procedure of device fabrication and *J–V* characteristics in a flux of 100 mW cm^−2^ simulating. The solar cells yielded a PCE of 2.0% with *V*_OC_ of 0.585 V, short‐circuit current density (*J*_SC_) of 5.9 mA cm^−2^ and FF of 0.58. When stretched to 18.5% for 11 full cycles, the devices still retained a PCE of ≈1.2%. Despite the moderate efficiency of the OSCs reported, the strategy paves a venue to make stretchable PV devices. We envision that highly efficient and stretchable OSCs will be realized through using the methods below: i) the usage of efficient photoactive layers with low band‐gap conjugated polymers, ii) the modification of PEDOT:PSS anodes for an electro‐optical trade‐off, and iii) interface shields for charge‐carrier transport and high device stability.

#### 6.2.2 Stretchable PV Foils Transferred on Prestrained Elastomeric Substrates

**Figure 26:**
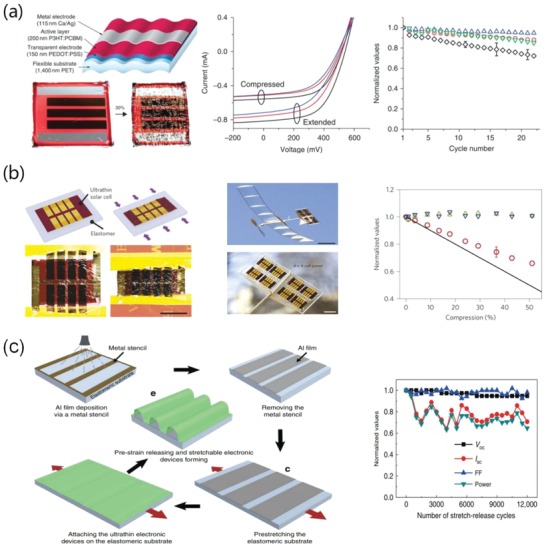
Stretchable PV devices. a) Stretchable OSCs with 1.4 µm PET. *I*−*V* characteristics are shown at 1 (black), 11 (red), and 22 (blue) cycles for the extended and 50% compressed states. The value of FF (green), *V*_OC_ (blue), *I*_SC_ (red), and power (black) under cycling test. Reproduced with permission.^165^ Copyright 2012, Nature Publishing Group. b) Stretchable PSCs, snapshot of the model plane during solar‐powered outdoor flight, and device performance versus uniaxial compression. *V*_OC_ (blue) and FF (green) remain unchanged, and *J*_SC_ (red) decreases as the active area shrinks. Reproduced with permission.^31^ Copyright 2015, Nature Publishing Group. c) Schematic diagram of stretchable device fabrication. OSC performance under a cyclic stretching at 20% strain. Reproduced with permission.^218^ Copyright 2018, Nature Publishing Group.

Stretchable optoelectronics based on periodic ordered buckling structures commonly show high mechanical compliance by retaining the buckling profiles in stretching–releasing tests. A unique transferring strategy enables stretchable PV devices, that is, by coating the foils of PVs onto a prestretched elastomeric stamp, it led to the formation of buckled or wrinkled configurations of PV foils. For instance, Kaltenbrunner et al. demonstrated ultrathin stretchable organic solar cells with PEDOT:PSS anodes. The stretchable devices were realized by transferring the ultrathin solar cells to a prestretched elastomer (**Figure** **26**a).^165^ The photovoltaic performances of the OSC devices are shown as follows: *V*_OC_ of 0.58 V, *J*_SC_ of 11.9 mA cm^−2^, FF of 61%, and PCE of 4.2%. In terms of device stretchability, the wrinkled devices survived quasi‐linear compression to below 70% of the initial area; with the cyclic compression and stretching to 50% at over 20 full cycles, the devices had marginal loss in efficiency and no visible defects beyond the external contact points.

The aforementioned transferring strategy is also suitable to fabricate stretchable, ultrathin, and lightweight PSCs. For instance, Kaltenbrunner et al. continued to demonstrate the stretchable PSC foils. Figure 26b shows the schematic diagram of the stretchable PSC foils with biaxial strains, and the extreme compliance of the ultrathin PSCs.^31^ The ultrathin PSC foils were transferred to a prestretched 3M VHB 4905 acrylic elastomer. Biaxial stretching was performed on a radial extender.^217^ Then, the foils were laminated to a prestretched elastomer. Upon a relaxation of the tape, a folding and wrinkled microstructure was formed and it allowed a variety of flexing and stretching deformations for the PSCs. Finally, the PSC foils were mounted on surfaces of radial extenders and had the ability to afford a radial compression down to 44% in area. The 2‐µm‐thick cells weigh only 4.6 g m^−2^ (5.2 g m^−2^ with PU) and have a power‐per‐weight of 26 W g^−1^ (23 W g^−1^ with PU). In light of the transferring strategy, Yin et al. reported a simple and universal technology to introduce ordered buckling structures into stretchable OSC and OLED devices (Figure 26c).^218^ The stretchable OSC held an outstanding mechanical robustness in 12 000 cycle stretching–releasing tests at 20% strain. It is believed that the transferring strategy is a universal method of making high‐efficiency and stretchable optoelectronics.

#### 6.2.3 Stretchable Mesh‐Shaped PV Fibers

Fiber‐like‐shaped PVs can be made into spring or mesh shapes. These configuration designs open a route to realize the stretchable PVs. For instance, Peng et al.^219^ reported a stretchable OSC that consisted of P3HT:PCBM photoactive layers, PEDOT:PSS (Clevios PH1000 mixed with 2‐propanol) HTLs and single walled CNT (called SWCNT) anodes. The *R*_sh_ of the PEDOT:PSS films was decreased via doing the polar protic solvent (e.g., 2‐propanol).^220^, ^221^ 2‐propanol minimized the square resistance of the films as low as 150 Ω sq^−1^. It is reckoned that the thick PEDOT:PSS layers (≈100 nm) incorporated with SWCNTs also served as a hybrid anode. The resulting OSCs yielded an initial PCE of 1.23%, retaining 1.19% efficiency in 100 cycle stretching–releasing tests at 30% strain. Besides, the PCE delayed by 12% when the devices were strained to 40%, mostly due to a decreased electrical conductivity of the PEDOT:PSS/SWCNT composites. It is envisioned that the fiber‐shaped PVs will enable wearable, portable, and elastic PV modules through woven them into textiles and clothing.

#### 6.2.4 Intrinsically Stretchable PVs

**Figure 27:**
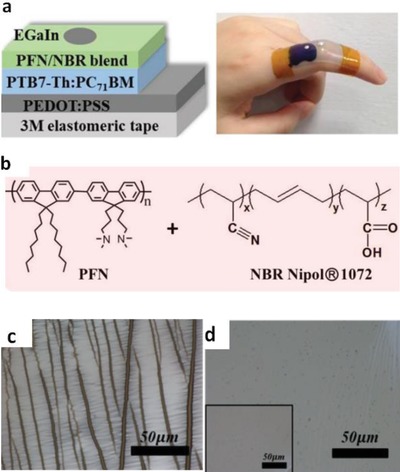
a) Stretchable OSCs with its real device photo attached onto the finger. b) Chemical structures of PFN and NBR. Optical microscopy images of the c) PFN and d) PFN/NBR films subjected to 60% strain. Reproduced with permission.^223^ Copyright 2017, American Chemical Society.

The stretchable organic solar cells mentioned above were not intrinsically stretchable because of the microscale buckle/wrinkle profiles employed to accommodate the tensile strains. Intrinsically stretchable optoelectronics require that all the components (a photoactive layer, the electrodes sandwiched it, and even interface buffer layers) are intrinsically stretchable. It has been challenged to fabricate this type of highly stretchable devices with satisfactory efficiency until now. Lipomi et al.^222^ ever reported the lowly stretchable OSCs (<5% elongation) on PDMS stamps. In terms of device fabrication, 5% DMSO and 1% Zonyl‐doped PEDOT:PSS was used as a stretchable anode, while the liquid metals of EGaIn were used as a cathode. However, the intrinsically stretchable OSCs commonly showed an unsatisfactory efficiency. It lagged far behind that of rigid devices fabricated on glass substrates. Hsieh et al.^223^ demonstrated a stretchable all‐polymer OSC comprising a PEDOT:PSS anode with DMSO and Zonyl treatments and an electron‐extraction layer (**Figure** **27**). The PCBM‐based device fabricated on 3M elastomeric tapes yielded a high PCE of 3.21% and it had an intrinsic stretchability of 5%.

All‐polymer blends are suitable to mechanically robust and intrinsically stretchable OSCs. Kim et al.^224^ found a stretchable photoactive layer incorporating polymer donors and polymer acceptors. The resulting all‐polymer OSCs exhibited a high PCE of 6.64% and rivaled the control flexible PCBM‐based devices with a PCE of 6.12%. The all‐polymer photoactive layers showed a superior mechanical durability and a considerable stretchability as compared to that of fullerene‐based photoactive layers, that is, the elongation at break and the toughness of the all‐polymer photoactive layers are over 60 times and 470 times higher than those of the fullerene‐based ones, respectively. It is mainly attributed to the higher intrinsically ductile property of the polymer acceptor and a better entanglement with the polymer donors. Although the configuration of the as‐fabricated devices belongs to flexible types rather than stretchable ones, the milestone research with the findings of highly stretching all‐polymer photoactive layers are energetically favorable for the future realization of intrinsically stretchable OSCs. We also advise that both plasticized PEDOT:PSS electrodes and all‐polymer photoactive layers are integrated together into intrinsically stretchable OSC foils that have a high probability of affording a large tensile strain (at least 10%) with a high efficiency retained. It is very promising for the CP applications into wearable and portable PVs with high efficiency and mechanical compliance. New methods to making highly elastic photoactive layers and intrinsically stretchable transparent electrodes are also urgently needed for the developments of stretchable optoelectronics.

### 6.3 Stretchable Organic Thin‐Film Transistors

One of the most fundamental organic electronic devices is the organic thin‐film transistor, because of the usefulness in establishing charge transportation characteristics of semiconducting materials. OTFTs comprising organic field effect transistors (OFETs) and organic electrochemical transistors (OECTs) have emerged as state‐of‐the‐art potentiometric sensing platform for making flexible, versatile and disposable biosensors, owing to their inherent capability of signal amplification.^225^, ^226^, ^227^, ^228^ Several approaches such as buckled/wrinkled strategies,^229^, ^230^ conducting materials integrated elastic meshes^231^, ^232^ and stretchable conductor wires^145^, ^201^ have been introduced to make the transistors amenable to tensile strains. Here, we mainly focus on the substantial applications of flexible and stretchable PEDOT:PSS films into stretchable OFETs as interconnectors (including electrodes) and stretchable OECTs as electroactive layers.

#### 6.3.1 Stretchable OFETs

A typical OFET is a three‐terminal device configuration that consisted of an organic semiconductor channel, a dielectric layer and electrodes (source, drain, and gate). Source/drain electrodes are connected intimately through the organic semiconducting layer, whereas the dielectric layer is deposited between the semiconductor and the gate electrode. When the gate voltage is applied on the OFET, charge carriers are generated and then accumulated at the interface between the semiconductor and dielectric layer, resulting in the formation of the conducting channels between source and drain electrodes. The channel current is well controlled by the applied gate voltage, demonstrating the signal amplification function of the OFETs.

**Figure 28:**
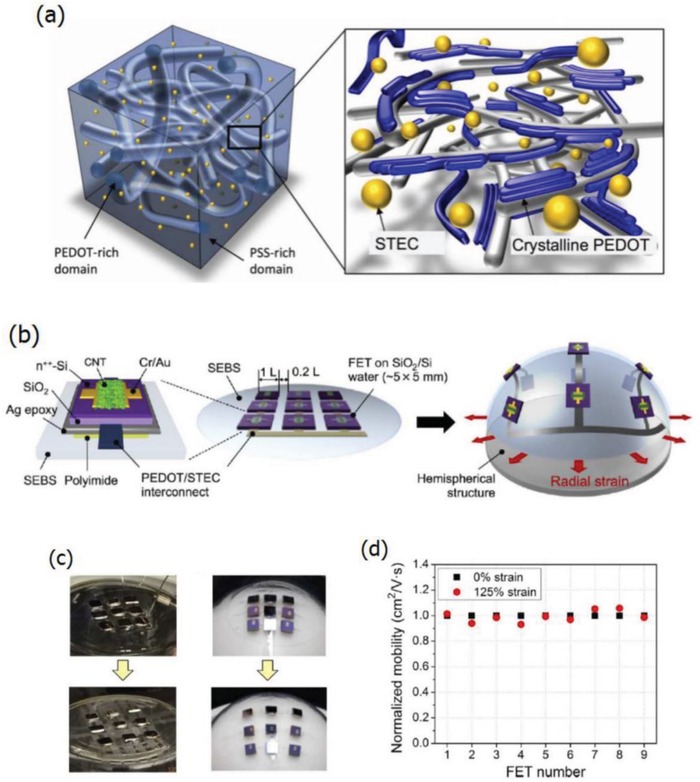
a) Schematic diagram of the typical PEDOT:PSS film with stretchable and conductive ionic additive enhancers (STEC). b) Schematic representation of a rigid‐island FET array with stretchable PEDOT:PSS interconnects. c) Photographs of the FET arrays that are strained in all directions on flat surfaces and spherical objects, respectively. d) Normalized mobility of individual devices when the array is stretched to 125% strains. Reproduced with permission.^109^ Copyright 2017, American Association for the Advancement of Science.

Chemical modification tends to improve the PEDOT:PSS conductivity and it enables a deformable PEDOT:PSS electrode or interconnector to fabricate stretchable OFETs. One approach is to take the advantage of rigid‐island matrices, which could keep both the high transistor performance from rigid devices and superior stretchability from PEDOT:PSS interconnectors. For instance, Bao et al. demonstrated a highly stretchable and conductive PEDOT:PSS interconnector incorporated with ionic additives used to make stretchable OFETs.^109^ The ionic additives consisting of sulfonate/sulfonimide anions served as effective dopants and induced a substantial enhancement in both conductivity and stretchability for the PEDOT:PSS films, as shown in **Figure** **28**a. Then, the rigid‐island OFET array (fabricated on silicon substrates) was integrated through the additive‐assisted PEDOT:PSS interconnectors (Figure 28b), demonstrating the capability of multidimensional stretching both on flat and spherical surfaces (Figure 28c). The mobility of the OFETs performed less than 10% variation even under 125% strain, illustrating a stabilized conductance held by the PEDOT:PSS interconnectors (Figure 28d). Besides, Jin‐Seo Noh demonstrated a method to enhance the stretchability of PEDOT:PSS films for making organic interconnects.^135^ A block copolymer poly(dimethylsiloxane‐*b*‐ethylene) (PDMS‐*b*‐PEO) was introduced as a mediator to improve the miscibility of PEDOT:PSS and PDMS, a transparent elastomer through its amphiphilic nature. The film blend performed conductivity comparable to pure PEDOT:PSS films and it showed a notable maximum strain up to 75%, which is promising for the CP applications into highly stretchable devices.

Other stretchable OFETs are demonstrated through using the PEDOT:PSS as electrodes. For example, Li et al. designed a healable stretchable transparent electrode via the hybridization of Ag NW networks and PEDOT:PSS.^233^ An ethanol‐water wetting process between Ag NW and PEDOT:PSS enabled a stretchable and healable hybrid conductor that showed a high conductivity (15 Ω sq^−1^) with 100% strain capability. More recently, Bao et al. reported an intrinsically stretchable and healable OFET in which PEDOT:PSS/carbon nanotube bilayer was employed as source and drain electrodes through a spray‐coating process.^234^ The mobility of the devices remained higher than 1 cm^2^·V^−1^·s^−1^ even under 100% strain, and the skin‐inspired stretchable OFETs were successfully fabricated and operated well under deformations, which was promising for wearable transistor applications. In summary, the stretchable PEDOT:PSS films have the combined advantages of high electrical conductivity and mechanical ductility, large‐size printing, good patternability, and biocompatibility, which are energetically favorable for making wearable, portable, and epidermal electronics.

#### 6.3.2 Stretchable OECTs

Organic electrochemical transistors are highly sensitive transducers that convert ionic signals into electrical ones. The devices are regarded as a promising platform for organic bioelectronics.^235^, ^236^, ^237^ An OECT is a typical transistor in which the channel current is modulated by the applied gate bias through the aqueous electrolytes. The ionic species in the electrolytes are driven to inject into the active channel layer accompanied with an electrochemical doping/dedoping process, thus inducing a high transconductance and high on–off ratio in the devices. So far OECTs have been employed as various kinds of chemical and biological sensors, including protein,^238^ ions,^239^ dopamine,^36^, ^240^ lactate,^241^ bacteria,^242^ and uric acid level and glucose level in saliva.^38^

**Figure 29:**
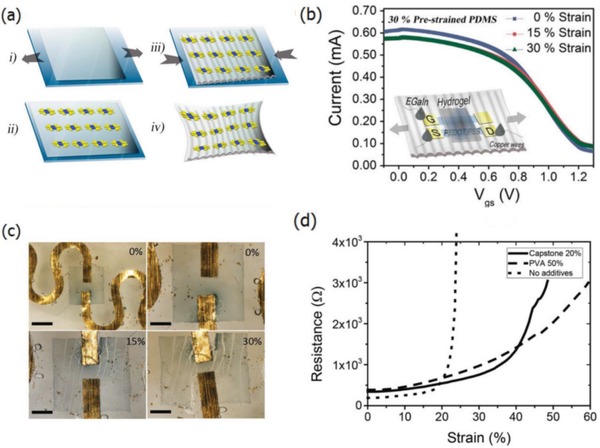
a) Fabrication process for stretchable OECT using parylene transfer and orthogonal patterning on 30% prestrained PDMS. b) Transfer characteristics of stretchable OECTs at 0%, 15%, and 30% strains. Reproduced with permission.^242^ Copyright 2017, American Chemical Society. c) Photographs of the channel after different strain processing. Scale bar is 1 mm for the top left picture and 500 µm for the others. d) Resistance change of PEDOT:PSS layer as a function of the strain, under different additive conditions (Capstone, PVA, or no additives). Reproduced with permission.^243^ Copyright 2018, Nature Publishing Group.

The first strategy for fabricating stretchable OECTs is based on the prestretched technique. Zhang et al.^243^ reported stretchable OECTs that were fabricated on 30% prestretched PDMS substrates through a transfer patterning of metal electrodes and an orthogonal patterning of PEDOT:PSS channel layer (**Figure** **29**a). Then a biocompatible “cut and paste” hydrogel was deposited to connect the gate electrode with PEDOT:PSS channels. As shown in Figure 29b, the transfer characteristics of the OECTs under different applied strains up to 30% performed relatively stable current modulation behavior, indicating that both the hydrogel electrolytes and the PEDOT:PSS channel could efficiently induce the reversible doping/dedoping processes under different stretched conditions. The work demonstrates a pathway of patterning microscale OECTs on stretchable substrates, which is significant for further conformable/implantable sensing applications.

Marchiori et al.^244^ demonstrated a fabrication process for patterning fully the stretchable OECT through the combination of laser ablated curved metallic interconnectors with additive enhanced PEDOT:PSS film. The active stretchable areas of the devices were connected with stretchable Au interconnectors, as shown in Figure 29c. The formulation of PEDOT:PSS channel was tuned by blending with either Capstone FS‐30 or PVA to enhance the film stretchability (Figure 29d), finally achieving the OECT device with a maximum stretchability of 38% while remaining high channel current (0.2 mA) and high transconductance (0.35 mS). The work is favorable for the development of OECTs onto living organs for continuous recording.

These OECT devices are able to transduce ionic current to electrical current for glucose sensing and cell monitoring,^245^, ^246^, ^247^ suggesting promising applications as bioelectronics. Notably, such transistors were realized through using a wrinkled device configuration or a curved metallic interconnection.^248^ We envision that the intrinsically stretchable transistors that operate well under >20% strains have a high probability of being realized through incorporating with Zonyl/Capstone (e.g, >5 vol%), ionic liquids and other derivatives.

**Figure 30:**
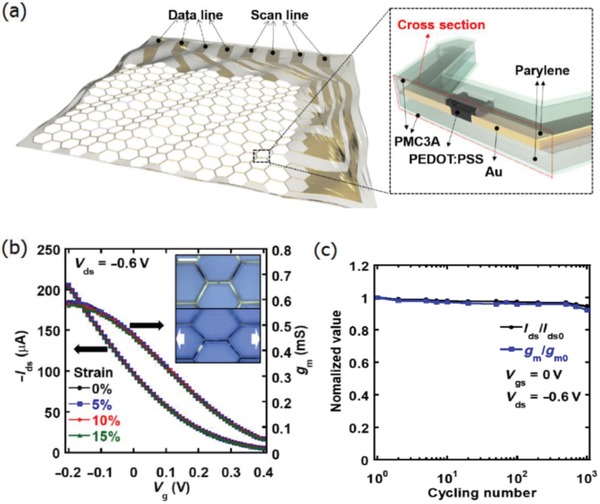
a) Image of the stretchable OECT array on a honeycomb grid parylene substrate. Insert: cross section of the OECT structure. b) Transfer and transconductance curves after 0%, 5%, 10%, and 15% stretching of the OECT array. Insert: Microscope image of the 10% stretched OECT array. Scale bar: 200 µm. c) Normalized channel current (*I*_ds_) and transconductance (*g*_m_) versus cycling numbers of 15% stretching. Reproduced with permission.^249^ Copyright 2018, American Association for the Advancement of Science.

Furthermore, a stretchable active OECT array for electroanatomical mapping was demonstrated by Someya's group in 2018.^249^ In the work, the honeycomb grid substrate was employed to integrate the PEDOT:PSS‐based OECTs, owing to its superior performance in both structural stretchability and mechanical stability (**Figure** **30**a). The transfer and transconductance curves were characterized under various stretching conditions (0%, 5%, 10%, and 15% strain), as shown in Figure 30b. Furthermore, the cyclic test was carried out for the OECT arrays under 15% extension strain. Figure 30c indicates that only 7% decrease was observed for channel current and transconductance value, demonstrating the excellent mechanical durability of the OECT integrated honeycomb grids. Finally, the novel stretchable OECT array was applied for high precision monitoring of high spatial and temporal resolution electrophysiological signal collection.

**Figure 31:**
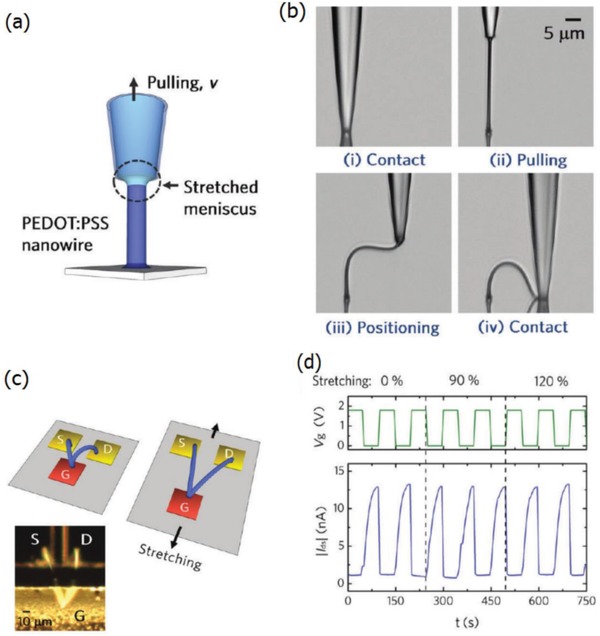
a) Schematic diagram of the fabrication process for 3D PEDOT:PSS nanoarches. b) Optical micrographs of the fabrication steps: i) contact, ii) pulling, iii) positioning, and iv) contact. c) Stretching of the OECT device with source (S), drain (D), and gate (G) electrodes. d) Channel current response to the periodic switching of gate voltage under different stretching conditions (0%, 90%, and 120%). Reproduced with permission.^250^ Copyright 2012, American Chemical Society.

Kim et al.^250^ reported an organic nanowire–based OECT through accurate 3D writing technique. It demonstrated an outstanding stretchability up to 270% without an obvious compromise in electrical performance. As shown in **Figure** **31**a, the PEDOT:PSS liquid meniscus was first created when the micropipette touched the substrate. As the micropipette was pulled up, the PEDOT:PSS nanowire was formed from the stretched meniscus. The nanowire was then precisely deposited on the patterned electrodes of OECT and further employed for stretchability test (Figure 31b,c). The switching behavior of the OECT with applied gate voltage pulse maintained stable under large stretching up to 120% strain (Figure 31d). The results suggest that the organic materials are promising to be integrated into novel stretchable nanodevices through the accurate 3D writing technique. However, stretchable OTFTs are still in the early stage and far behind the practical implementations.^251^ The device performance is closely related to the electrical property and mechanical strength of the CP materials and dielectric materials. The device stability against oxygen, humidity, ultraviolet light, and heat should be considered with respect to the organic transistor applications, because the as‐cast CP materials are vulnerable to being water‐swelled, oxidation and photodegradation when exposed in the air.

## 7 Summary and Outlook

**Figure 32:**
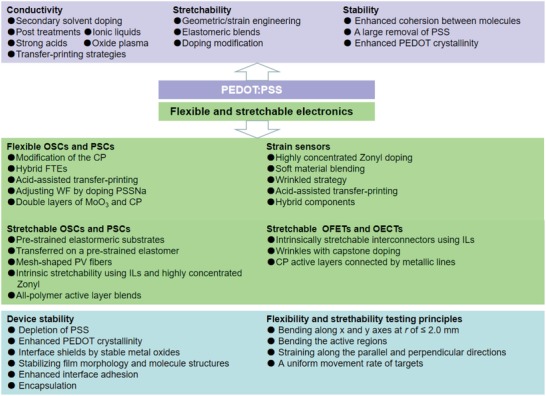
Schematic diagrams of the methods and strategies for the PEDOT:PSS modifications and their developments of flexible and stretchable devices.

Wearable, flexible, and stretchable devices come to the forefront of organic electronics and are needed in wide range of applications as stated above. Much effect has been devoted to the PEDOT:PSS films with high performances (i.e., electrical conductivity, mechanical compliance, and stability) for augmenting the practical implementations. PEDOT:PSS films exhibit the striking merits such as a variety of electrical characteristic among insulators, semiconductors, and conductors, a change of mechanical property among rigidity, flexibility, stretchability, and elasticity as well as good thermal stability. **Figure** **32** summarizes an overview of the review addressing the key points of PEDOT:PSS films and these flexible and stretchable devices. The methods of device stability enhancements and mechanical testing principles are presented as well. However, there are several questions that remain open in the research including device stability, device integration, and mechanical flexibility.

First of all, aqueous PEDOT:PSS dispersions are acidic at pH 1, and the PSS probably diffused into other components of devices followed by detrimental reactions, thereby deteriorating the device performances including efficiency, mechanical compliance, and stability. The underlying mechanisms of performance degradation are ambiguous yet. For boosting these device performances, the effective pathways are advised including the large removal of both PSS and strong acid residuals from the PEDOT:PSS matrices, the interface shields by compact and stable metal oxide layers (i.e., Cr_2_O_3_, MoO_3_, and vanadium pentoxide [V_2_O_5_]), and the usage of dopants for stabilizing the phase‐segregated morphology and molecule structures. All that methods are expected to be energetically favorable for boosting the stability of the flexible and stretchable electronics.

Second, in the case of flexible and stretchable device fabrication, it is highly desirable to use the types of components all that show a considerable flexibility and intrinsic stretchability. For example, it is of grand potential to the realization of highly efficient and stretchable organic solar cells via employing the intrinsic stretchable PEDOT:PSS electrodes, stretchable CP buffer layers, liquid metals and all‐polymer photoactive layers with high tensile modulus and elongation at break. Besides, we advise to establish a standard testing method for effectively evaluating the flexibility and stretchability of the optoelectronic devices. For an effective flexing test, the devices should be bent along *x* and *y* axis, respectively, at small radii of no more than 2.0 mm. Note that the data coming from a uniaxial bending test was not accurate and even incorrect; and the active region (i.e., the whole top electrodes rather than the photoactive layers surrounding the electrodes) should afford the bending stresses and mechanical deformations. For stretching tests, the devices need to be tensile strained along the parallel and perpendicular directions, respectively, for at least 1000 cycles at a uniform movement rate we advised. The real metrics of device performance versus mechanical cycles/bending radii are obtained in light of the relatively scientific measurements.

Third, the interface adhesion between the CP and its underlying substrates should be considered with respect to the electrode preparation and device integration. Unlike the strong interactions between as‐sputtered ITO products and rigid glass substrates on bottom, the adhesion between CP films and their underlying plastic substrates are indeed weak. For instance, the interface interaction between as‐cast PEDOT:PSS films and plastic substrates was as low as 20 N m^−1^; upon the strong acid modification, the interface interaction was reduced to 5.3 N m^−1^.^80^ As a result, the modified CP films were prone to peeling off from the plastic substrates when undergoing mechanical insults such as shearing, bending, twisting and tensile strains. It is thus suggested that via inserting a transparent layer of cross‐linkable polymers or highly adhesive materials into the interfaces between PEDOT:PSS and underlying substrates, it enables a durable device that affords a variety of mechanical insults.

Finally, encapsulation is urgent and significant for evolving the stability of flexible and stretchable devices. Cracks, wrinkles, and defects of both device components and encapsulation materials were generated in harsh flexing and strain‐loading tests along with geographical deformations. Air permeation across the encapsulated layers and around them probably occurs. Besides, the package layers had a probability of destroying the underlying device components due to mechanical abrasion. Thus, it is of grand challenge to encapsulate the whole devices toward a long‐term air stability. Emerging package materials and technologies are urgently needed for the developments of the flexible and stretchable devices. For moving forward with the commercial applications of the wearable, flexible, and stretchable electronics, early stages for the satisfactory encapsulation are a key step.

We envision that the conclusions and perspectives mentioned above will encourage a fast transition of the next‐generation flexible and stretchable electronics from lab‐scale studies to industrial‐scale implementations.

## Conflict of Interest

The authors declare no conflict of interest.
